# Inflammasome activation dictates the efficacy of antimycobacterial activity of frontline TB drugs

**DOI:** 10.1371/journal.ppat.1014384

**Published:** 2026-07-16

**Authors:** Anjali Singh, Kanika Bisht, Garima Maurya, Nidhi Yadav, Ranjan Nanda, Vivek Rao

**Affiliations:** 1 CSIR-Institute of Genomics and Integrative Biology, New Delhi 110025, India; 2 Academy of Scientific and Innovative Research (AcSIR), Ghaziabad, Uttar Pradesh 201002, India; 3 Translational Health Group, International Centre for Genetic Engineering and Biotechnology (ICGEB), New Delhi 110 067, India; New Jersey Medical School, UNITED STATES OF AMERICA

## Abstract

Recent developments in tuberculosis (TB) treatment have identified an enormous potential of host-directed therapies (HDT)) in achieving better and faster control of infection. We have previously demonstrated the synergistic effect of sertraline (SRT) with frontline TB drugs in clearing infection in murine tissues. Our attempts to uncover the mechanistic basis of this enhancement, using sertraline as a probe, help identify host signalling pathways critical for controlling *Mycobacterium tuberculosis* (Mtb). We identify a significant role for sertraline-mediated modulation of mitochondrial physiology and consequent reactive oxygen species (ROS) generation as a secondary signal, leading to greater IL-1β release and K+ efflux from macrophages via NLRP3 inflammasome activation. We thus highlight an important relationship between mitochondrial physiology and inflammasome activation, enabling infected macrophages to better control Mtb.

## Author summary

Tuberculosis (TB), caused by *Mycobacterium tuberculosis*, remains a leading cause of infectious disease mortality worldwide and requires prolonged multidrug treatment. The emergence of drug resistance and the complexity of host–pathogen interactions highlight the need for targeted host-directed therapies (HDTs) as a promising approach to improve treatment outcomes. In this study, we have investigated the basic mechanisms that allow the use of sertraline, a widely used antidepressant, as an adjunct to standard TB therapy. Our findings reveal that sertraline activates macrophage NLRP3 inflammasome, a key component of the innate immune response. By specifically altering mitochondrial function, thereby increasing reactive oxygen species (ROS) production, SRT enhances inflammasome-mediated release of the pro-inflammatory cytokine IL-1β and promotes potassium efflux, processes that strengthen macrophages’ ability to control intracellular Mtb growth. Together, our results uncover a previously underappreciated link between mitochondrial physiology and inflammasome activation during TB infection.

## Introduction

The current trends towards TB control have centred on harnessing host responses to bacterial control as an effective strategy to circumvent drug-associated development of resistance in the population [1–4]. Consistent with these efforts, we have designed an adjunct TB therapy that significantly enhances Mtb growth control and improves host survival by adding the FDA-approved antidepressant drug SRT to the current regimen [5]. We have also established the importance of the NLRP3 inflammasome and the resultant production of IL-1β in the enhanced bacterial control by SRT. Given the important role of inflammasome activation in controlling macrophage innate defence against infections such as Mtb [6–8], we sought to understand the molecular basis of SRT-mediated inflammasome activation. Here, we reveal the significant role of the NLRP3-dependent Gasdermin D (GSDMD) in both cellular and in vivo models of infection, thereby establishing the importance of this previously unclear signalling axis in the control of Mtb by SRT [6,7]. In line with previous reports of a two-step activation cascade for inflammasomes [8–11], we uncover mitochondrial perturbation and the resultant ROS in SRT-treated cells as the secondary trigger working downstream of the Mtb infection-induced activation of immune signalling. We further deposit the efflux of K^+^ ions consequent to GSDMD activation as a key factor in hampering the pathogen-beneficial type I IFN response in Mtb infection, thus highlighting a novel and important role for mitochondrial physiology in the antibiotic-mediated control of infection.

## Methods

### Ethics

Human subjects: The study was conducted in strict accordance with recommendations of the National Ethical Guidelines for Biomedical and Health Research Involving Human Participants, Indian Council of Medical Research (ICMR), Government of India. The protocols followed were approved by the Institutional Human Ethics Committee of the Institute of Genomics and Integrative Biology, proposal no. 10, 2016, and Ref. no. CSIR-IGIB/IHEC/2017–18 Dt. 08.02.2018. Written patient-informed consent was obtained prior to commencing the work. Animal work was carried out in accordance with the requirements of the institutional animal ethics committee, with approval (IGIB/IAEC/10/Nov/2023/05).

THP-1 dual monocytes were cultured in complete RPMI 1640 (with 2 mM L-glutamine, 25 mM HEPES, 50 µM sodium pyruvate, and 10% FBS) and differentiated into macrophages with PMA (Phorbol 12-myristate 13-acetate; 100nM) for 24h. Human monocyte-derived macrophages (MDMs) were generated from PBMCs of healthy volunteers by the addition of 50ng/mL of GM-CSF (Granulocyte-Macrophage Colony-Stimulating Factor) for 7 days. Mtb Erdman was grown in Middlebrook 7H9 medium with 4% Albumin-dextrose-saline or Middlebrook 7H10 plates supplemented with 10% oleic acid-albumin-dextrose-catalase (OADC) at 37 °C. Log-phase Mtb cultures were washed twice with PBST (1x PBS with 0.05% Tween 80) and centrifuged at 800g for 10 minutes to generate a single-cell suspension, which was used to infect macrophages for 6 hours at an MOI of 5. Macrophages were treated either 30 minutes before infection or prior to treatment with the various inflammasome inhibitors, such as MCC950 (10µM), VX-765 (20µM), IHC-2 (20µM), DSF (disulfiram; 10µM), DMF (dimethyl fumarate; 50µM), and KCl (Potassium chloride; 50mM). For treatment of cells with LPS and nigericin, only a pre-treatment with LPS (100 ng/mL) for 3h and nigericin (10 µM) for 45 minutes was given prior to infection. Following this, cells were either left untreated or treated with antibiotics Isoniazid (H-20 ng/mL) and Rifampicin (R-100 ng/mL), with or without SRT (20 µM), and the bacterial numbers were estimated by serial dilution plating on Middlebrook 7H10 agar plates. Cytokine expression in cell supernatants was analysed by ELISA. The luminescence of the cell supernatants after addition of the substrate (QUANTI-Luc, Invivogen) was used to quantify the type I IFN responses in THP1 dual cells. GSDMD expression was detected by immunoblotting with a monoclonal antibody according to recommended protocols. Mitochondrial ROS was measured in cells treated with 5 µM Mitosox for 30 min and imaged on an Invitrogen EVOS M5000 Imaging System at different time points. The mitochondrial potential was evaluated in cells stained with 1 µM TMRE (Tetramethylrhodamine ethyl ester perchlorate) at 37 ºC for 30 minutes by flow cytometry. For macrophage infection studies, isoniazid (H), rifampicin (R) - HR (H-20 ng/mL and R-100 ng/mL) or HRS with SRT 20 µM were used. For *in vivo* experiments, drugs were administered *ad libitum* in drinking water containing 1% sucrose after 4 weeks of infection. The treatment regimen included: 1- HRZE H (100 mg/kg), R (40 mg/kg), pyrazinamide (Z, 150 mg/kg), ethambutol (E, 100 mg/kg), 2- HRZES with SRT at 10 mg/kg, 3- HRZED and 4- HRZESD with disulfiram at 300 mg/kg. BALB/c mice (aged 6–8 weeks) were infected with Mtb by aerosol delivery of ~ 500 cfu per animal in a dedicated ABSL-3 facility. Lungs were used for bacterial count estimation and histological examination.

For measuring cellular potassium levels, two methods were employed: 1) ICPMS (iCAPTM TQ ICP-MS, Thermo Scientific, USA) of cell lysates. Following stimulation of macrophages for 3h in isosmotic buffer K or buffer W, with and without 50mM KCl, cells were incubated in the dark in 150µL of 70% HNO_3_ and 50 µL of 30% H_2_O_2_ for 10 min and then digested with HNO_3_ and H_2_O_2_ (ramp = 250ω for 10 min, Hold = 250ω for 5 min and cool = 55 ºC). A standard curve was used to estimate the concentration of K^+^ ions in the samples. 2) Staining with a fluorescent potassium-specific dye- IPG-4 AM (ION Potassium Green – 4 Acetoxymethyl ester) in 0.5% (w/v) pluronic acid for 1h at 37 ºC and imaged on the Invitrogen EVOS M5000 Imaging System.

Data was analyzed for statistical significance by the student t-test with Welch’s correction or one-way ANOVA for parametric data and the two- tailed Mann-Whitney test or Kruskal-Wallis test for non-parametric data.

The detailed protocol (S1 File) and reagents (S1 Table) used in the study are given as a supplementary information file.

## Results

### Inflammasome activation is critical for enhanced antibiotic efficacy in Mtb infection

**Fig 1:**
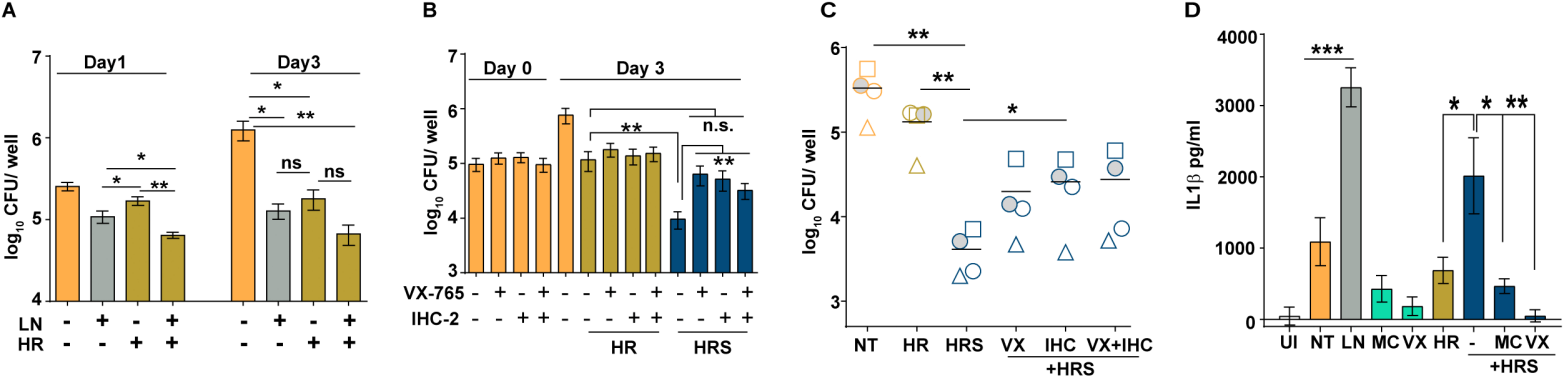
Sertraline-mediated enhancement of antibiotic efficacy is dependent on inflammasome activation. Cells were infected and either left untreated (NT) or treated with HR, HR+ sertraline (HRS) alone or in combination with the inflammasome inhibitors, caspase 1 (VX765- VX)/ NLRP3 (MCC950- MC). Bacterial numbers at day 1 and day 3 or day 5 (panel C) post-treatment are represented as mean CFU + SEM of triplicate assays from 3 independent experiments (N = 3). A) The effect of LPS and nigericin (LN) on bacterial growth in THP1 macrophages. B, C) Bacterial growth in Mtb-infected THP1 (B) or human monocyte-derived macrophages (C). Each symbol in panel C represents a sample from one healthy individual. D) The levels of secreted IL-1β in culture supernatants of Mtb-infected THP1 macrophages were measured after 24h of infection by ELISA and are represented as mean concentrations (pg/ml) ± SEM from triplicate assays in N = 3 experiments.

While the activation of the host cell inflammasome in controlling Mtb infections is undebatable, studies have revealed both host-beneficial and pro-pathogenic outcomes of this process [12–14]. Our previous study has hinted at an important role for the host cell inflammasome in augmenting control of Mtb when sertraline is combined with frontline TB drugs [5]. As an initial step to investigate whether inflammasome activation determine antibiotic efficacy, we observed that adding LPS and nigericin (LN) to HR significantly decreased bacterial burden by ~2.7-fold compared with HR alone (4-fold vs 1.5-fold w.r.t. untreated), corroborating a synergistic effect of inflammasome activation on antibiotic efficacy. In fact, the addition of LN alone was sufficient to restrict Mtb growth by 3-fold very early in infection (day 1), which increased to >10-fold in the LN group compared to the untreated groups by day 3 (Fig 1A).

We have previously shown that the effect of SRT in augmenting Mtb control can be reversed by the inhibition of the NLRP3 inflammasome by MCC950 [15]. To investigate the complete cascade of inflammasome activation, we tested the effect of sertraline on bacterial control by inhibiting the associated caspase with VX765 or IHC-2, specific inhibitors of caspase 1 and caspase 4, respectively. While SRT alone enhanced HR-mediated control of Mtb in macrophages by 6.5-fold on day 3, the addition of inhibitors, despite not altering the initial uptake, completely reversed this increase, with bacterial numbers reaching levels comparable to those in HR-treated macrophages (Fig 1B). This inhibition of caspase was also observed in primary human monocyte-derived macrophages, in which both IHC-2 and VX765 reversed the heightened bacterial control observed in HRS-treated samples across all individual samples, suggesting the importance of caspase/inflammasome activation (Fig 1C).

Inflammasome activation results in the secretion of mature IL-1β and IL18 from the activated macrophages [16]. Infection with Mtb induced significant levels of IL-1β in macrophages (~1000pg/mL). While treatment with HR did not alter IL-1β secretion, the addition of SRT induced ~2-fold higher IL-1β secretion in macrophages (Fig 1D). In line with previous reports, IL-1β levels in macrophages treated with LPS and nigericin were ~3-fold higher than in infection alone. Both the inflammasome inhibitors MCC950 and VX765 significantly reduced IL-1β secretion, further supporting a dominant role for inflammasome activation in SRT’s effect.

### Sertraline-induced gasdermin D activation is responsible for higher IL-1β secretion in macrophages

**Fig 2:**
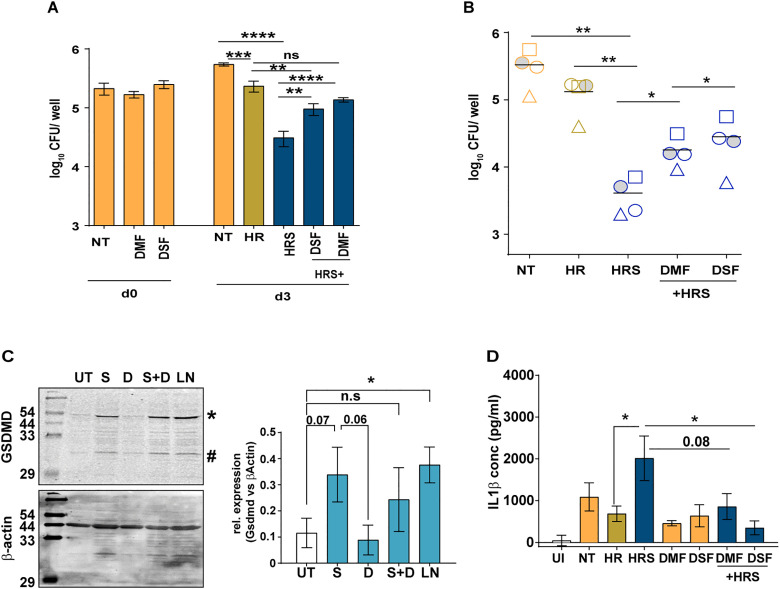
Sertraline activates gasdermin D in macrophages. Cells were infected with Mtb and either left untreated (NT) or treated with HR, HR+ sertraline (HRS) alone or in combination with gasdermin D activation inhibitors- dimethyl fumarate (DMF)/ disulfiram (DSF). A, B) Bacterial growth in THP1 (A) or monocyte-derived macrophages (B). Each symbol in panel B represents a sample from one healthy individual. Bacterial numbers at day 0 and day 3 post-treatment (A) and at day 5 (B) are represented as mean CFU + SEM of triplicate assays from 3 independent experiments (N = 3). The values for NT, HR, and HRS are the same as those used in Fig 1B, since the experiments were conducted at the same time. C) The expression levels of gasdermin D after 24h of treatment (premature form- *) in the crude lysates of infected macrophages on treatment with LPS + nigericin (LN), SRT or DMF. The amount of the mature N-terminal form (#) was analysed by immunoblotting, quantified by densitometric analysis, and is represented as fold-normalised to β-actin from N = 3 independent experiments. β-actin was used as a control (lower panel). D) The levels of secreted IL-1β in culture supernatants of Mtb-infected THP1 macrophages. The values for NT, HR, and HRS are the same as those used in Fig 2D, since the experiments were conducted at the same time. The level of IL-1β after 24h of infection, measured by ELISA, is shown as mean concentrations (pg/ml) ± SEM from triplicate assays in N = 3 experiments.

Typically, induction of the NLRP3 inflammasome leads to the formation of a GSDMD pore, releasing pro-inflammatory cytokines such as IL-1β and IL18 [6]. Macrophages treated with specific GSDMD inhibitors - dimethyl fumarate (DMF) or disulfiram (DSF), along with HR or HRS, were tested for the ability to restrict bacterial growth. Despite a negligible impact on the initial uptake of Mtb (d0), the addition of DSF or DMF reduced the effect of sertraline in augmenting HR-dependent bacterial control. In comparison with a significant increase in bacterial control by SRT in combination with HR compared with HR alone, treatment with DSF or DMF reversed this effect: DMF completely abrogated the SRT-dependent increase, while DSF supported a partial but significant reversal by 3–4-fold (Fig 2A). A similar pattern of 2–4-fold reversal in bacterial control was evident in primary human monocyte-derived macrophages, with the addition of either DSF or DMF supporting a dominant role for SRT in activating GSDMD (Fig 2B). The importance of GSDMD activation by proteolytic cleavage was further corroborated by the increase of the 31kDa N- terminal active form of GSDMD in cells treated with either SRT or LPS+ Nigericin (LN), which was completely absent in cells treated with DMF (D) (Fig 2C). Interestingly, treatment of human MDMs with SRT induced cell swelling and the formation of large, fluid-filled vacuolar structures, as previously documented with GSDMD activation in macrophages [17,18] (S1A Fig). This effect was also completely reversed by the addition of DSF, implicating the action of SRT in activating host cell inflammasomes via the GSDMD signaling pathway. In line with the determinant role of GSDMD in the release of IL-1β from activated macrophages, HRS alone induced 2.5-fold higher IL-1β secretion than either the untreated or HR-treated infected macrophages. In contrast, this increment was completely abolished by the addition of DSF/ DMF (Fig 2D).

### GSDMD activation is critical for increased antibiotic efficacy

**Fig 3:**
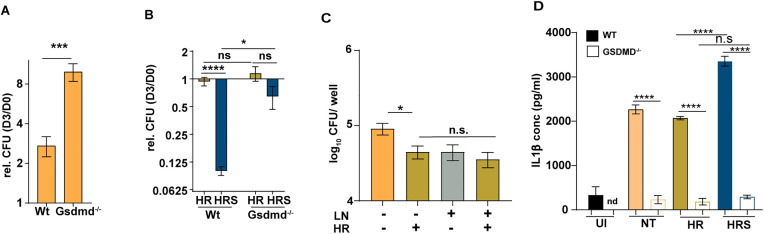
Gasdermin D activation is critical for Mtb control by infected macrophages. A, B, C) Bacterial growth in Mtb-infected, Wt or GSDMD-deficient THP1 macrophages. (NT- A)- Untreated cells or (B)-treated with HR, HRS or (C)- pretreated with the inflammasome activator LN. Bacterial numbers on day 3 relative to day 0 are represented as mean CFU + SEM of triplicate assays from 3 independent experiments (N = 3). D) The levels of IL-1β secreted from Wt or GSDMD-deficient THP1 macrophages after 24h of infection by ELISA and are represented as mean concentrations (pg/ml) ± SEM of triplicate assays from 2 independent experiments (N = 2).

In order to definitively implicate the role of GSDMD in bacterial control by macrophages, we tested the effect of SRT in GSDMD^-/-^ macrophages. Consistent with previous reports [19–21], Mtb-infected GSDMD^-/-^ macrophages were impaired in their ability to restrict bacterial growth harbouring 4–5-fold higher bacteria than wildtype (Wt) cells alone or on treatment with HR by day 3 of infection (Fig 3A). However, despite similar HR efficiency in controlling Mtb, the efficacy of SRT was significantly altered across the two macrophage lines. GSDMD-deficient cells did not significantly enhance bacterial growth control, whereas the 10-fold reduction in bacterial numbers observed in the GSDMD-sufficient cells was not seen in the GSDMD-deficient cells (Fig 3B). Moreover, the increased control observed with the LPS and nigericin-induced inflammasome activation in HR-treated Wt macrophages (Fig 1A) was lost in the GSDMD^-/-^ macrophages (Fig 3C). The importance of the inflammasome-GSDMD signalling axis in bacterial control was further validated by the significantly lower levels of IL-1β in response to infection at 18h of treatment in the GSDMD^-/-^ macrophages, resulting in the loss of significant differences in cytokine secretion between HR and HRS-treated macrophages (Fig 3D).

### GSDMD is critical for *in vivo* control of Mtb

**Fig 4:**
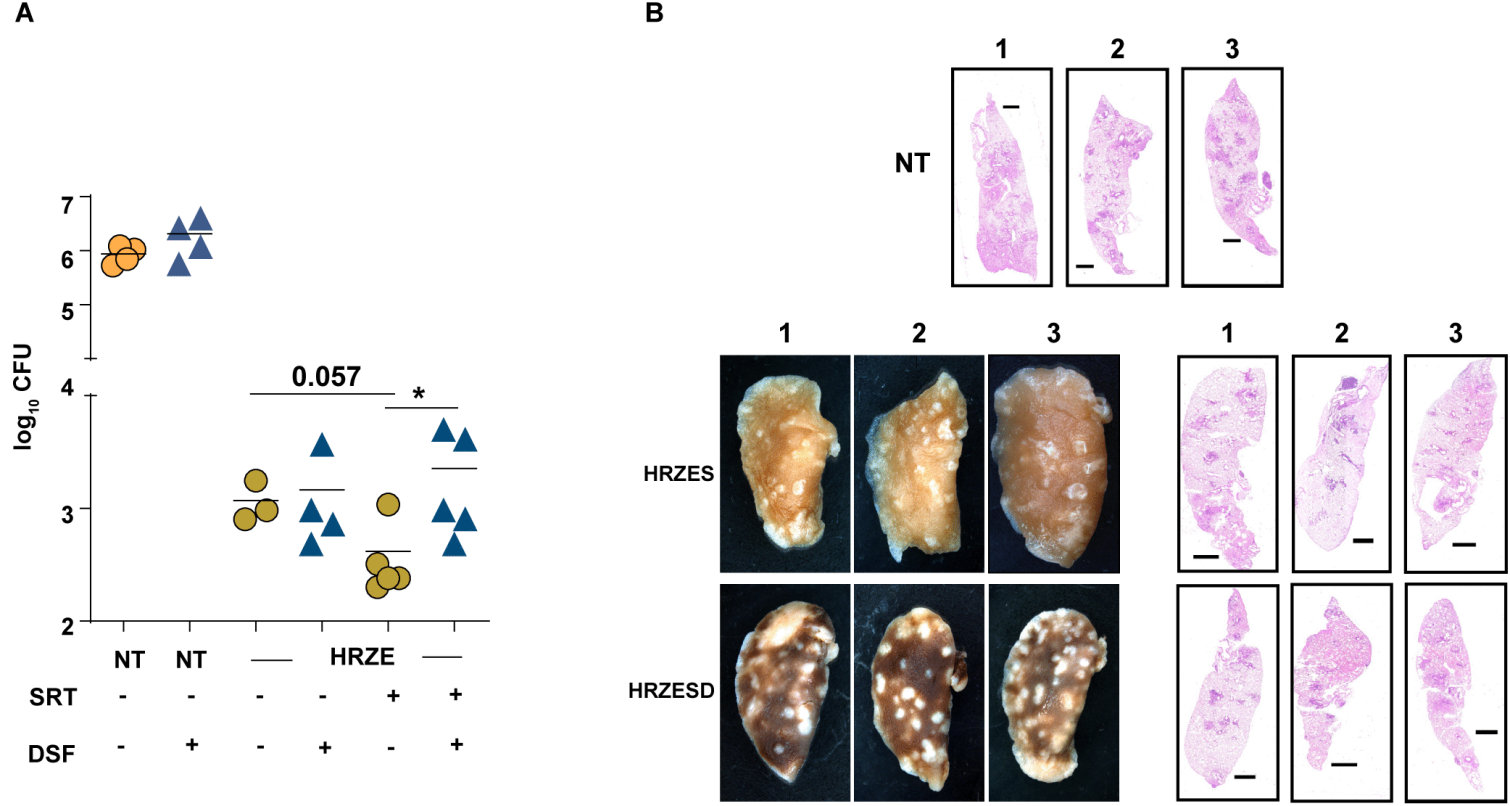
In vivo Gasdermin D activation dictates Mtb control. A) Bacterial growth in lungs of Balb/c mice that were infected with 500 cfu of Mtb by aerosol and either left untreated (NT) or treated with HRZE, HRZE+ sertraline (HRZES) alone or in combination with disulfiram (D, HRZED, HRZESD). Bacterial numbers at 4 weeks after treatment were enumerated by CFU plating and are presented as the mean CFU ± SEM from 4 or 5 mice per group. B) Macroscopic images of lung lobes from HRZES + DSF-treated animals and histological examination of lung sections stained with H&E from NT – DSF and HRZES ± DSF-treated animals. Scale bars denote 1mm.

To test the relevance of GSDMD *in vivo*, Balb/c mice were infected with Mtb and treated with antibiotics and the GSDMD inhibitor, disulfiram. The addition of disulfiram did not significantly alter the Mtb growth kinetics alone or in combination with frontline TB drugs (HRZE) (Fig 4A). However, the effect of DSF treatment was pronounced in HRZES-treated animals; a complete reversal of the SRT-dependent increase in Mtb control (as seen in HRZES) in the lungs was achieved by including DSF, resulting in bacterial numbers similar to those in HRZE-treated animals. Moreover, this inhibitory effect of DSF was also evident in tissue damage in animal lungs (Fig 4B). As expected, H&E-stained histopathological analysis of the lungs from HRZES-treated animals showed fewer microscopic lesions compared to untreated or HRZE-treated animals. However, sections from animals treated with HRZES and DSF showed significantly more lesions, similar to those in the HRZE-treated or untreated groups (Fig 4B).

### SRT-mediated potassium efflux regulates Mtb-induced type I IFN responses and is critical for enhanced bacterial control

**Fig 5:**
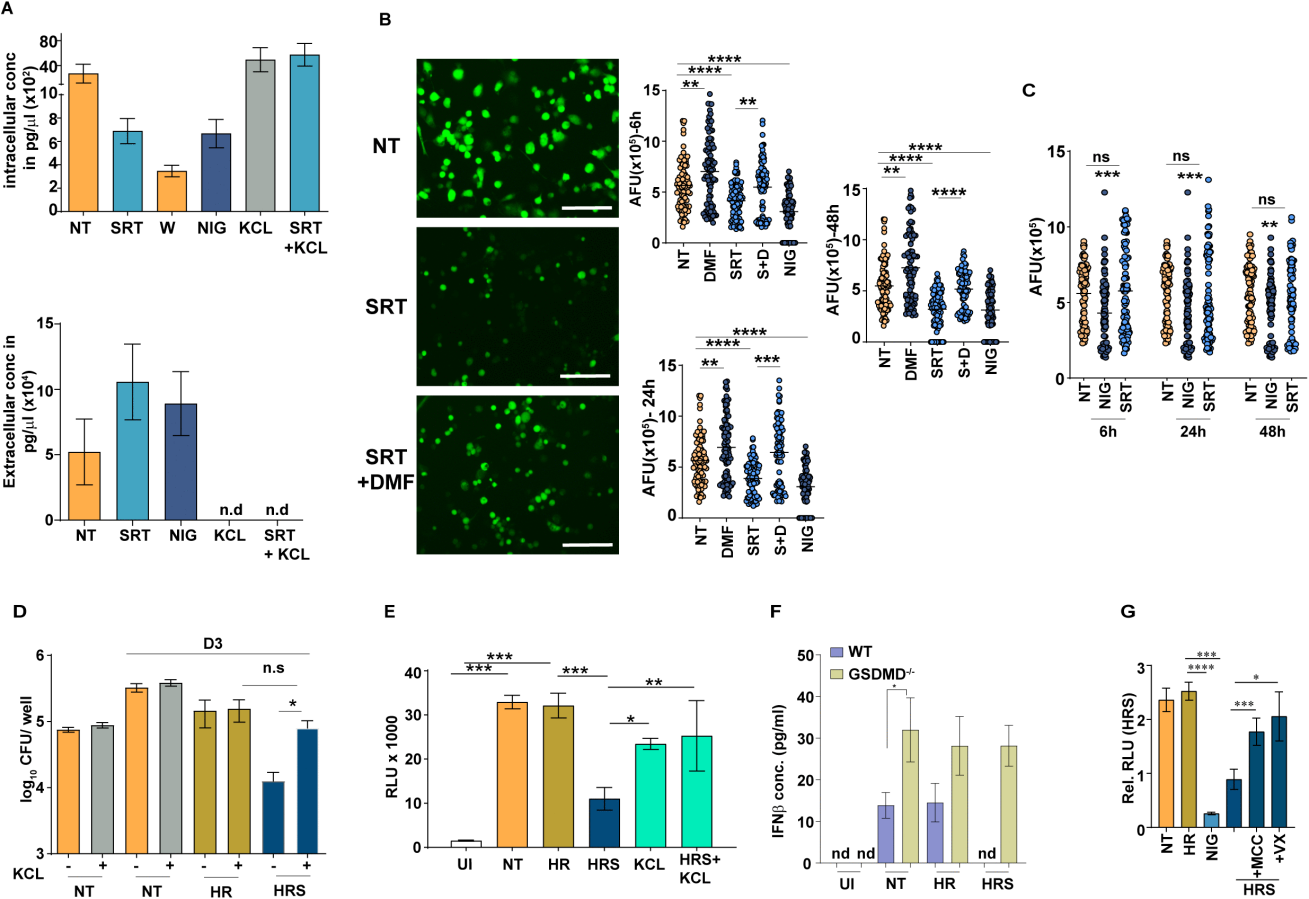
Sertraline-induced potassium efflux is critical for enhancing mycobacterial control. A) The levels of potassium were estimated in macrophages and in the cell supernatants by ICP-MS after 3h of treatment. B, C) Estimation of potassium in macrophages as depicted by dye binding in Wt (B) or GSDMD^-/-^ (C) macrophages. Representative images of macrophages stained with IPG-4AM are shown, while the fluorescent intensities at 6, 24, and 48h post-treatment are graphically represented as mean arbitrary fluorescence units (AFU) +SEM of 90 cells for 3 independent experiments. D) The effect of KCl treatment on the ability of SRT to enhance bacteria growth control in THP1 macrophages. Bacterial numbers at day 3 post-treatment were enumerated by CFU plating and are represented as the mean CFU ± SEM across triplicate assays from 3 independent experiments (N = 3). Mtb-infected macrophages, Wt or GSDMD^-/-^, cells were left untreated or treated with SRT, with and without KCL or nigericin (NIG), or with DMF. E-G) The effect of inflammasome activation on the type I IFN response in control (UI) or Mtb-infected THP1 macrophages. E) Effect of K+ efflux inhibition with KCL, F) in Wt or GSDMD-/- macrophages, G) in the presence of inflammasome activator- nigericin (NIG) or inhibition (MCC950 and VX765-VX). The extent of type I IFN-regulated luminescence elicited at 24 h post-infection (p.i.) is shown as relative units (RLU) + SEM of triplicate assays from three independent experiments (N = 3). Cells were either untreated (NT) or treated with HR alone or in combination with the indicated molecules.

With an active efflux of intracellular potassium ions, a consequence of gasdermin D activation [22], we investigated the relevance of this efflux in macrophages following SRT treatment. Akin to cells incubated in a potassium ion-depleted buffer (W), resulting in a 6–7-fold decrease in intracellular potassium, treatment with SRT, as well as nigericin, facilitated significant K^+^ efflux from macrophages, leading to 4–5-fold lower ionic concentrations in comparison to untreated cells (Fig 5A). This release was reflected as a significant increase in K^+^ ion levels in the supernatants of cells treated with SRT or nigericin. Furthermore, the addition of KCl, despite abolishing K^+^ efflux in SRT-treated cells, completely failed to alter the untreated cells. To examine the kinetics of K^+^ flux in macrophages, cells were stained with the fluorescent potassium indicator IPG-4 AM and imaged after treatment. Again, in both the SRT and nigericin-treated cells, significantly lower fluorescence of IPG-4 AM was observed as early as 6h post-treatment compared to untreated cells (Fig 5B). Inhibition of GSDMD activation by DMF completely blocked this efflux, restoring the fluorescence in SRT-treated macrophages to levels comparable to those of untreated cells. This pattern was maintained even after 24h and 48h of SRT treatment in the GSDMD^+/+^ macrophages as opposed to the complete failure of GSDMD^-/-^ macrophages to extrude K^+^ ions following SRT treatment at any time of the experiment (Fig 5C). Further, the importance of K^+^ efflux in bacterial control was validated by the complete loss of extended bacterial control upon exogenous KCl supplementation specifically during HRS treatment of macrophages (no effect on bacterial control in HR-treated or untreated cells; Fig 5D). Given the contrasting regulation of K^+^ efflux and type I IFN signalling in macrophages [22], we tested the effect of modulating SRT-dependent K^+^ extrusion on the Mtb-induced type I IFN response. As expected, HRS restricted macrophage type I IFN signaling by ~6–8 fold in comparison to Mtb infection alone or with HR by 24h in macrophages, a property that was overturned by the addition of KCL (Fig 5E). Loss of GSDMD in macrophages resulted in significantly greater IFN secretion from Mtb-infected cells, regardless of treatment, including HRS (Fig 5F). Further, the inhibition of the inflammasome by MCC950 or VX786 significantly offset the restricted type I IFN response of HRS-treated cells, with the complete restoration of the type I IFN levels in the HRS-treated cells to untreated levels, supporting the role for SRT-mediated inflammasome activation in the type I IFN inhibition (Fig 5G).

### The SRT-driven mitochondrial ROS level in macrophages is critical for inflammasome activation

**Fig 6:**
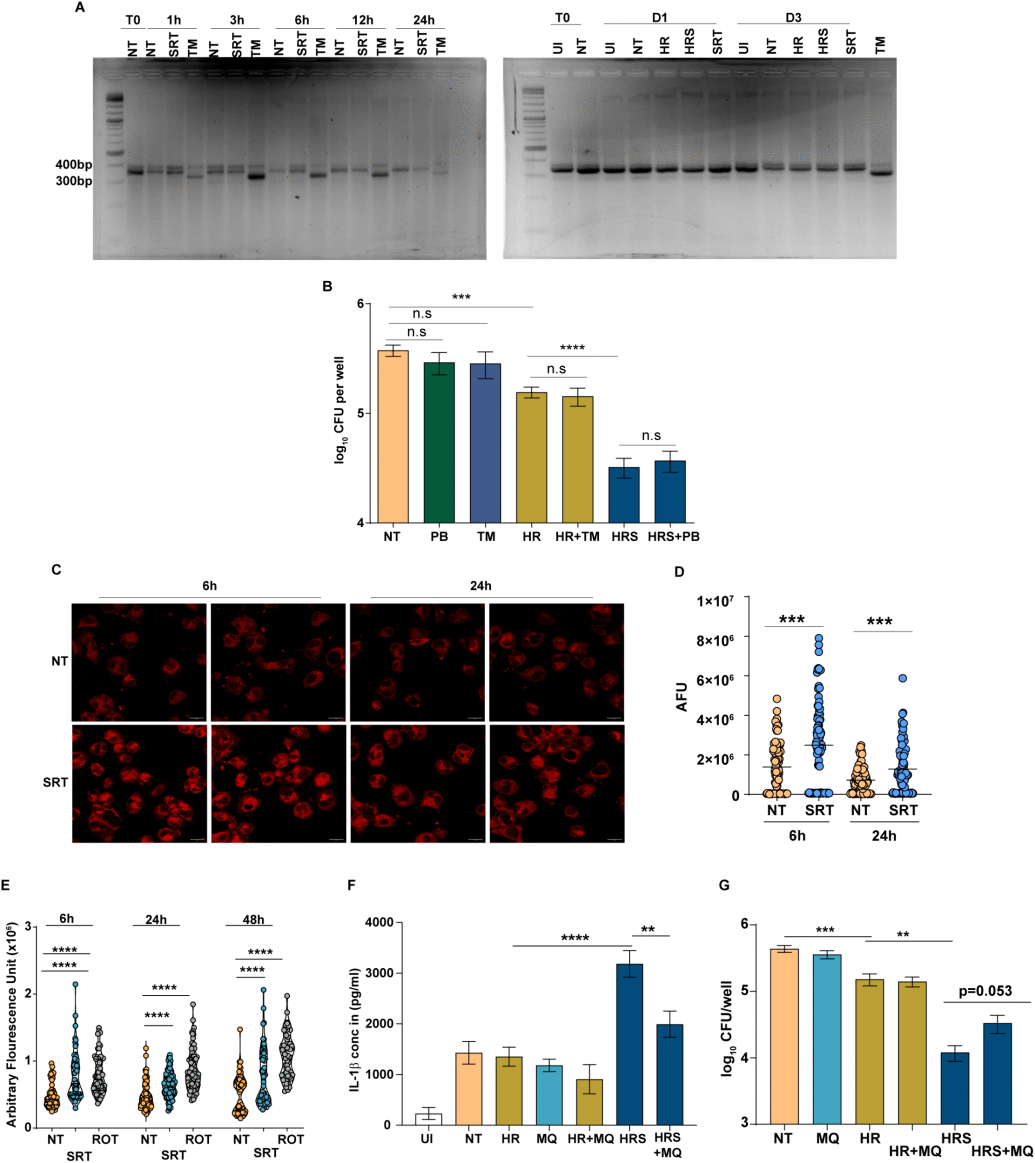
Sertraline induces a significant alteration in the mitochondria of macrophages. A) The effect of sertraline on ER stress was analyzed by estimating the extent of XBP-1 splicing in uninfected (left panel) or the Mtb infected (right panel) macrophages and left untreated (NT) or treated with HR, SRT or HRS or tunicamycin (TM) for the indicated time intervals. B) The effect of modulating ER stress in cells by TM or 4-phenylbutyric acid (PB) on bacterial control. Bacterial numbers in Mtb-infected cells, either left untreated (NT-B) or treated with HR or HRS, on day 3 post-treatment, are represented as mean CFU ± SEM of triplicate assays from 3 independent experiments (N = 3). C, D) The extent of mitotracker deep red staining in untreated or SRT-treated macrophages was analysed by microscopy. The mean arbitrary fluorescence units were calculated and are graphically represented as multiple cells/fields from 3 independent experiments at the indicated time points (N = 3). E) The levels of mitochondrial ROS induced in THP1 macrophages following treatment with SRT were estimated by staining with MitoSox. ROS values were quantified as mean AFU ± SEM across multiple cells/images, as depicted graphically from three independent experiments (N = 3) at the indicated time points. F-G) The effect of the addition of mitoquinone (MQ) on the levels of IL-1β secretion in Mtb-infected macrophages at 24h (F) and bacterial control at day 3 (G) are represented. IL-1β levels are depicted as mean concentrations (pg/ml) ± SEM of triplicate assays from 3 independent experiments (N = 3). Bacterial numbers in Mtb-infected cells, either left untreated (NT-F) or treated with HR or HRS for 3 days, are represented as mean CFU ± SEM of triplicate assays from 3 independent experiments (N = 3).

While Mtb infection could prime the Inflammasome activation in macrophages, organellar dysfunction (ER or mitochondria) could provide the secondary signal [23,24]. Given that ER stress actively triggers XBP-1 splicing, we evaluated cleaved XBP-1 RNA levels in cells treated with SRT or tunicamycin (TM), a potent ER stress inducer. While tunicamycin induced near-complete splicing of XBP-1 as early as 1h with a gradual increase until 24h after treatment, SRT or Mtb infection alone or in combination with HR or HRS failed to activate this process at any point of treatment (Fig 6A). Additionally, the absence of any observed effect of modulating the ER stress by tunicamycin (activator) or 4-phenylbutyric acid (PB, inhibitor) on the efficacy of HR or HRS further negated this process in the SRT-mediated bacterial control (Fig 6B). In contrast, an examination of macrophages treated with SRT revealed significantly elongated mitochondria compared with untreated cells, suggesting a possible association between SRT-dependent changes in mitochondrial physiology and inflammasome activation (S1B Fig). In fact, mitochondria in SRT-treated cells showed a significant increase in mitotracker deep red staining, which is dependent on the mitochondrial membrane potential, indicating that SRT significantly alters mitochondrial membrane potential (Fig 6C and 6D) [25]. This alteration of mitochondrial membrane potential was evident on increased TMRE staining of SRT-treated cells as early as 3h, indicating that SRT induces mitochondrial hyperpolarisation (S1C Fig). Mitochondrial physiology is a cellular hub linking cellular metabolism and the response kinetics of innate immune cells; significant ROS production is associated with alterations in mitochondrial physiology [26,27]. To evaluate the consequences of mitochondrial changes, we measured mitochondrial ROS levels using the ROS-specific probe MitoSox in SRT-treated macrophages. In contrast to the low levels of staining in untreated cells, SRT, like the potent mito-ROS inducer, rotenone, significantly increased ROS as early as 6h, and this effect persisted even after 48h of treatment (Fig 6E). Further, inhibition by mitoquinone (MQ), a mitochondria-specific ROS scavenger, affected HRS-dependent inflammasome activation and bacterial control in macrophages. Mitoquinone significantly reversed HRS-induced increase in IL-1β release in macrophages, restoring these levels to those observed in untreated or HR-treated cells (Fig 6F). Furthermore, the addition of mitoquinone partially accounted for the increased bacterial growth observed in HRS-treated cells, without altering the ability of HR-treated or untreated cells to control bacteria, highlighting the pivotal role of mitochondrial perturbation in inflammasome activation and the SRT-dependent amplification of bacterial control (Fig 6G).

## Discussion

Therapies that leverage host signalling pathways provide alternatives that circumvent the development of drug-resistant bacterial strains in the population [3]. We previously demonstrated that adding sertraline, by restricting the type I IFN response, enhanced bacterial control in preclinical models [5]. The study also revealed a possible role for inflammasome activation in this process. To understand this further, we used sertraline as a probe and identified the complete signalling cascade underlying inflammasome activation in macrophages, thereby uncovering a novel mechanism to increase the antimycobacterial activity of frontline TB drugs. In this study, we have defined the complete activation cascade of the inflammasome machinery by SRT using multiple inhibitors. None of the inhibitors induced toxic effects in the cells, even after 3 days of stimulation (S1D Fig). The toxic effects of LPS and nigericin are only observed by day 3 and not on day 1, further supporting an active role for inflammasome activation-mediated exacerbated release of IL-1β and the associated decrease in bacterial numbers.

Our study defines the role of SRT in activating inflammasomes without any significant cell death, with the assay using LPS & nigericin, as supplementary evidence for the role of inflammasome activation in controlling infection.

Specifically, we establish that the SRT-mediated inflammasome activation, culminating in active K^+^ ion efflux, is a key regulator of type I IFN responses in macrophages. Inhibiting this efflux significantly enhances the infection-dependent type I IFN response, substantially eroding the antimicrobial effect of the combination of sertraline and frontline TB drugs. The marked increase in IFN-β expression in SRT-treated macrophages following treatment of exogenous KCL to inhibit K^+^ efflux verifies that IFN-I signalling is indeed sensitive to changes in K⁺ flux and the cross-regulation of K⁺ efflux with type I IFN response in Mtb-infected macrophages. These findings are in support of a previous study that showed that Gasdermin D-mediated K⁺ efflux suppresses cGAS–STING activation and limits type I IFN responses [28].

Activation of caspase-1 and caspase-4 is crucial in inflammasome activation, and their inhibition leads to poor bacterial control [29]. Despite the combined inhibition of the two caspases, we did not observe a complete reversal of bacterial control in the HRS-treated macrophages. It is plausible that the activation of caspase-8 in Mtb infection could also mediate the GSDMD cleavage, potentially compensating for the loss of caspase-1 and caspase-4 activity [30].

Mtb infection has been associated with the induction of IL-1β via the NFκB pathway, which has been implicated in improved Mtb killing, both directly and through alternate effector functions [31,32]. Membrane pores created by GSDMD activation and oligomerisation function primarily in the release of mature IL-1β from live innate cells [33–35]. Recently, GSDMD has been shown to have an affinity for cardiolipin, enabling its binding to and permeabilisation of the bacterial cell membrane, leading to bacterial killing in the cytosol or within the phagosomes of phagocytes [36]. Additionally, GSDMD has been connected to targeting and eliminating cell-free bacteria when released from pyroptotic cells [37]. In fact, several intracellular bacterial infections initiate the GSDMD-mediated pyroptosis [38–40]. We convincingly demonstrate the importance of SRT-mediated GSDMD activation in Mtb control in infected macrophages using specific inhibitors such as DSF and DMF, as well as GSDMD^-/-^ deficient cells. While DSF, in addition to its well-established inhibitory activity on GSDMD activation, also constrains aldehyde dehydrogenase, modulates redox/Nrf2 pathways, and affects other cellular processes, DMF also reversed SRT-mediated control. Additionally, we have confirmed that biochemical inhibition of GSDMD by DSF and DMF significantly reduced secreted IL-1β in macrophages, a process dependent on the inflammasome. As further validation, GSDMD^-/-^ macrophages phenocopy the chemical inhibition, demonstrating reversal of SRT’s effect on bacterial growth control. Taken together, these complementary pharmacologic and genetic data strongly support the interpretation that loss of GSDMD activity impairs bacterial control in our model, while acknowledging that DSF may also affect other host pathways.

A recent study has identified infection-triggered GSDMD activation, leading to excessive necrosis, as an important aspect of Mtb pathogenesis [41]. In a separate study, Mtb was shown to play a potent role in triggering NLRP3-mediated pyroptosis, resulting in plasma membrane damage and the release of K^+^ ions from macrophages, which has been associated with bacterial spread [14]. Although GSDMD can permeabilize and kill bacteria, chronic GSDMD activation results in excessive inflammation, leading to tissue damage and cell death by pyroptosis [42]. However, given the high MOI (20) used, the lower MOI (5) used in our study argues for a novel mechanism of significant proteolytic activation of GSDMD by SRT that reorients phagocytic metabolism toward an alternative non-lytic state with increased IL-1β secretion and minimal necrosis, as evidenced by the insignificant levels of LDH release, in contrast to pyroptotic lysis [5]. This is in sync with recent evidence that supports the existence of two distinct states of GSDMD-activated macrophages: 1) a pyroptotic state characterised by cell lysis and 2) a hyperactivated state similar to the treatment with SRT, with minimal cell lysis and increased levels of GSDMD membrane pores allowing the release of mature IL-1β and other inflammatory mediators. This type of inflammasome-mediated host defence has been demonstrated to facilitate control of another intracellular bacterial infection (*Salmonella typhimurium*), allowing neutrophils to secrete IL-1β without compromising the integrity of the cell membrane, which is damaged during pyroptosis [43].

We also describe SRT’s ability to induce significant perturbations in mitochondrial physiology. This finding is particularly significant, given the emerging recognition of mitochondria as key regulators of innate immunity. Mechanistically, SRT-induced mitochondrial ROS can facilitate multiple pathways in inflammasome assembly and activation, such as the release of oxidised mitochondrial DNA, externalisation of cardiolipin, or the interaction of NLRP3 with TXNIP [44–46]. Recent studies have also suggested a direct interaction between GSDMD and mitochondria, leading to activation of host cell inflammasomes [36]. While we provide insights into the possible role of host cell cholesterol flux modulation by SRT as an upstream effector of mitochondrial impairment [47], the present study effectively links mitochondrial dynamics to immune regulation in TB.

Taken together, our study confirms the importance of inflammasome amplification in enhancing host defence mechanisms against Mtb, highlighting the critical link between mitochondrial function, inflammasome signalling, and pathogen clearance. A comprehensive understanding of the synergistic effects of inflammasome activation and antibiotic treatment would enable the screening for potential modulators and inhibitors as putative effective adjunct therapies for tuberculosis and improving host defence mechanisms.

## Supporting information

S1 Fig Data supporting the manuscript are represented as panels in the Figure. (TIF)

S2 Fig A pictorial representation of the major observations and conclusions of the study. Created in BioRender. Rao, V. (2026) https://BioRender.com/5a658fd.
(TIF)

S1 File Detailed methods used in the study are given. (DOCX)

S1 Table The reagents used in the study are given. (XLSX)

S1 Data Immunoblots of GSDMD activation. (ZIP)

S2 Data Monitoring kinetics of K+ efflux in sertraline treated macrophages. (ZIP)

S3 Data Gross lung images of Mtb infected and treated animals. (ZIP)

S4 Data Evaluation of mitochondrial ROS in macrophages treated with sertraline. (ZIP)
