# Supplementary material for: Inflammasome activation dictates the efficacy of antimycobacterial activity of frontline TB drugs
Source: PLoS Pathog. 2026 Jul 16;22(7):e1014384. doi: 10.1371/journal.ppat.1014384 (PMC13399518; doi:10.1371/journal.ppat.1014384)
Supplement: S1 File — (DOCX) [file ppat.1014384.s003.docx]

**S3 text: Detailed Methods**

**Culture of bacteria and cell lines.**

THP-1 monocytes were cultured in RPMI 1640 supplemented with 2 mM L-glutamine, 25 mM HEPES, 50 µM sodium pyruvate, and 10% fetal bovine serum at 37^0^C in a 5% CO₂ humidified incubator. PMA (Phorbol 12-Mysristate 13-acetate- P8139, Sigma Aldrich, USA) was used for differentiation into the macrophages for 24h. Human monocyte derived macrophages (MDM) were derived from PBMCs isolated from healthy volunteers by addition of 50ng/mL of granulocyte-macrophage colony-stimulating factor (GM-CSF) (Invitrogen) for 7 days in accordance with Institutional human ethics committee approval (Ref no: CSIR-IGIB/IHEC/2017–18). The macrophages were then removed from the plates and used for infection studies. For infections, Mtb Erdman was cultured in Middlebrook 7H9 broth (BD, Difco), supplemented with 0.5% glycerol, 4% Albumin-Dextrose-Saline (ADS), and PBST (0.05% Tween 80) at 37^0^C. Bacterial numbers were calculated in the single cell suspensions (SCS) and used to infect macrophages at MOI 5 6h. Following this, cells were either left untreated or treated with antibiotics (H-20ng/ml and R-100ng/ml) with and without SRT- 20µM and the bacterial numbers were estimated by plating serial dilutions of cell lysates on Middlebrook 7H10 agar containing 0.5% glycerol and 10% Oleic acid-Albumin-Dextrose-Catalase (OADC) supplement (HiMedia Laboratories, India) and incubation at 37^0^C for 3-4 weeks.

**Treatment of the Inflammasome modulators.**

Macrophages were pre-treated for 30 minutes with inhibitors, MCC950 (10µM), VX-765 (20µM), IHC-2 (20µM), DSF (10µM), DMF (50µM), and KCl (50mM) followed by infection with Mtb. For the activation of inflammasomes, cells were treated with 100ng/ml LPS for 3h and with nigericin for 45 min prior to infection with Mtb.

**Analysis of macrophage responses.**

The expression of cytokines in the cell supernatants was analysed by specific ELISA according to the manufacturer’s recommendations (IL1β, TNFα, BD OptEIA - BD biosciences), IFNβ (R&D systems). The levels of IFNβ were estimated in the THP1 dual cells by quantitating the luminescence of the cell supernatants following the addition of the substrate- QUANTI-Luc (Invivogen, France), as per the manufacturer’s recommendations. GSDMD expression was detected by immunoblotting with a monoclonal antibody HPA04487 (Prestige Antibodies® Sigma-Aldrich, USA) and fluorescently labelled secondary antibodies and visualized with a LI-COR Odyssey imaging system. For estimation of mitochondrial ROS**,** SRT-treated cells were incubated with 5 μM of the specific dye**-** MitoSOX for 30 min or rotenone at 10 μM and imaged in the Invitrogen™ EVOS™ M5000 Imaging System. For analysing the mitochondrial potential, SRT treated macrophages were stained with 1µM tetramethylrhodamine (TMRE) at 37 ºC for 30 minutes and analyzed by FACS (BD Accuri™ - BD Biosciences). CCCP at 10 μM was used as a control for the study.

**Mtb infection in mice model of infection**

All animal infections were conducted in a dedicated ABSL-3 facility as per the accepted recommendations of the IAEC (IGIB/IAEC/10/Nov/2023/05). BALB/c mice (aged 6-8 weeks) were infected with Mtb using an inhalation exposure system (Glas-Col, USA) for aerosol delivery of ~ 500 cfu per animal. Treatment with antibiotics and SRT was initiated after 4 weeks of infection and provided *ad libitum* in the drinking water containing 1% sucrose twice a week. Mice were administered the TB drugs (H-100 mg/kg, R-40 mg/kg, Z-150 mg/kg, E-100 mg/kg), with or without SRT (10 mg/kg) and in the presence or absence of DSF (300 mg/kg). At specific time points, animals were euthanized and the tissues were used for estimation of the bacterial numbers and histological examination. The formalin-fixed left caudal lung lobes were used for gross examination by a Zeiss (Semi 2000®C) bright field microscope and histological evaluation by H&E staining of sections in an Olympus microscope.

**Analysis of cellular potassium levels.**

Intracellular or excreted potassium levels were estimated in SRT-treated THP1 cells by two methods-

1. Mass spectrometric estimation in cells treated with SRT in isosmotic buffer K (130mM NaCl, 7mM Na_2_HPO_4_, 3mM NaH_2_HPO_4_. 5mM KCl, pH 7.4), buffer W- with 50mM KCl or without, for 3h were resuspended in 150µl of 70% HNO_3_ and 50 µl of 30% H_2_O_2_ for incubation for 10 min in the dark. Following digestion with HNO_3_ and H_2_O_2_ (ramp = 250ω for 10 min, Hold = 250ω for 5 min and cool = 55 ºC), the samples were filtered and analyzed in the ICP-MS machine (iCAPTM TQ ICP-MS, Thermo Scientific, USA). A standard curve was used to estimate the concentration of K^+^ ion in the samples.
2. staining with a fluorescent potassium-specific dye- IPG-4. For dye-based analysis, cells were treated with the dye in 0.5% (w/v) pluronic acid F-127 (Sigma-Aldrich, USA) for 1h at 37 ºC in a CO_2_ incubator in the dark and then imaged using the Invitrogen™ EVOS™ M5000 Imaging System.

**Cell Viability Assay**

Cell viability in the presence of the various inhibitors used in the study was determined using the MTT (3-(4,5-Dimethylthiaxol-2-yl)-2,5-Diphenyltetrazolium Bromide) assay, and the reagent was acquired from Sigma-Aldrich (M5655-1G). THP1 dual cells were seeded and differentiated in a 96-well plate at a density of 0.1 x 10^6^ cells/well. The cells were treated with inhibitors, including MCC950 (10 µM), VX-765 (20 µM), IHC-2 (20 µM), DSF (10 µM), DMF (50 µM), and KCl (50 mM) for 7 h in a humidified CO_2_ incubator. For LPS and Nigericin treatment, the cells were treated with LPS (100 ng/mL) for 3 h, followed by LPS and nigericin (10 µM) for 45 minutes, after which the cells were incubated in fresh media.

The MTT assay was performed at time points 1, 4, and 7 h during the treatments and at 24, 72, and 96 h post-treatment. For the assay, fresh media (without FBS) containing MTT reagent at 0.5 mg/mL was added to the cells, which were incubated for 1 h in a humidified CO2 incubator. Following incubation, MTT-containing media were carefully removed to avoid disturbing the formed formazan crystals, which were then dissolved in DMSO (150 µL/well) by light shaking for 2-5 minutes at room temperature. The absorbance of each well was measured at 565nm using a Tecan Infinite 200 plate reader. The experiment was performed in triplets and repeated three times. The cytotoxic effect of each inhibitor was expressed as a percentage of viable cells compared to the vehicle control.

**Statistical tests and significance**:

All statistical analyses were conducted using GraphPad Prism software. An unpaired t-test or multiple t-tests were applied to data pooled from two to three independent experiments each performed in triplicate. For parametric data analysis, the student t-test with Welch’s correction method or ordinary one-way ANOVA for multiple comparisons was used to determine the significance of the t-tests. For non-parametric data analysis statistical analysis was performed using a two-tailed Mann-Whitney test or Kruskal-Wallis test for multiple comparisons.

**Ethics**

Human subjects: The study was conducted in strict accordance with recommendations of the National Ethical Guidelines for Biomedical and Health Research Involving Human Participants, Indian Council of Medical Research (ICMR), Government of India. The protocols followed were approved by the Institutional Human Ethics Committee of the Institute of Genomics and Integrative Biology, proposal no. 10, 2016, and Ref. no. CSIR-IGIB/IHEC/2017-18 Dt. 08.02.2018. Written patient-informed consent was obtained prior to commencing the work. Animal work was carried out in accordance with the requirements of the institutional animal ethics committee, with approval (IGIB/IAEC/10/Nov/2023/05).

**Author contribution**

AS, KB and VR were instrumental in the design of the work. AS and KB were involved in conducting the work. NY and RN were involved in the design and conduct of the ICPMS based detection of potassium ions in cells. None of the authors have any conflict of interest.

**Acknowledgements**

This work was supported by the Council of Scientific and Industrial Research grants MLP2106 and MLP2012 to VR. CSIR-STS0016 is acknowledged for the continuous maintenance of BSL3 and ABSL2 facilities. CSIR- BSC0403 is duly acknowledged for the microscopy facility. The CSIR-India student fellowships (AS, KB, and GM) are acknowledged. The funders had no role in the study design, data collection and analysis, and preparation of the manuscript. Biorender.com is duly acknowledged for the preparation of the graphical abstract. The authors thank Ms Nikita Bhor, Ms Riya Sahu, and Mr Lakshay Kumar for proofreading and providing suggestions to improve the manuscript.
