# Supplementary material for: Inflammasome activation dictates the efficacy of antimycobacterial activity of frontline TB drugs
Source: PLoS Pathog. 2026 Jul 16;22(7):e1014384. doi: 10.1371/journal.ppat.1014384 (PMC13399518; doi:10.1371/journal.ppat.1014384)

## Slide 1
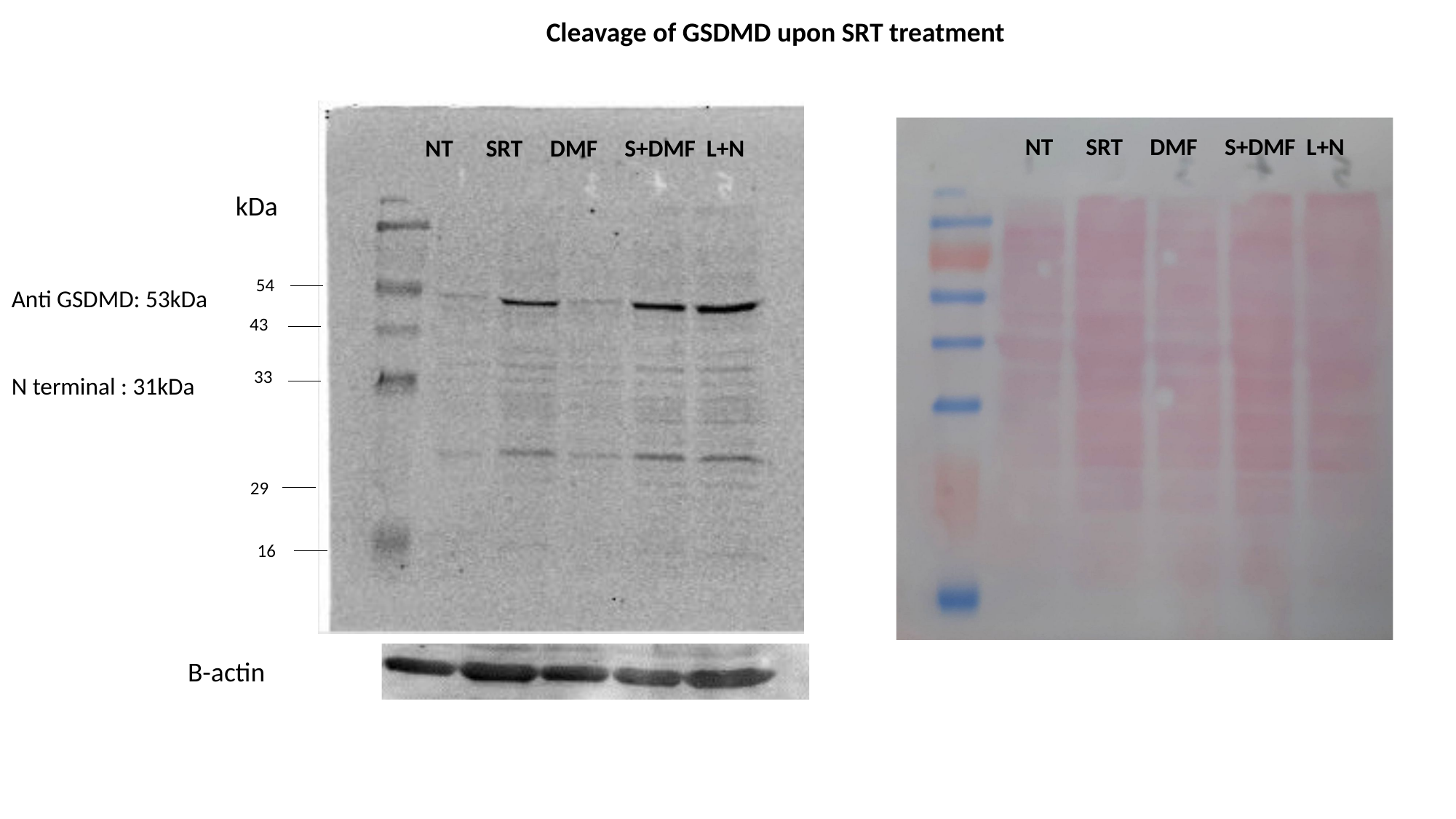

Cleavage of GSDMD upon SRT treatment
 NT SRT DMF S+DMF L+N
 NT SRT DMF S+DMF L+N
kDa
54
Anti GSDMD: 53kDa
N terminal : 31kDa
43
33
29
16
B-actin

## Slide 2
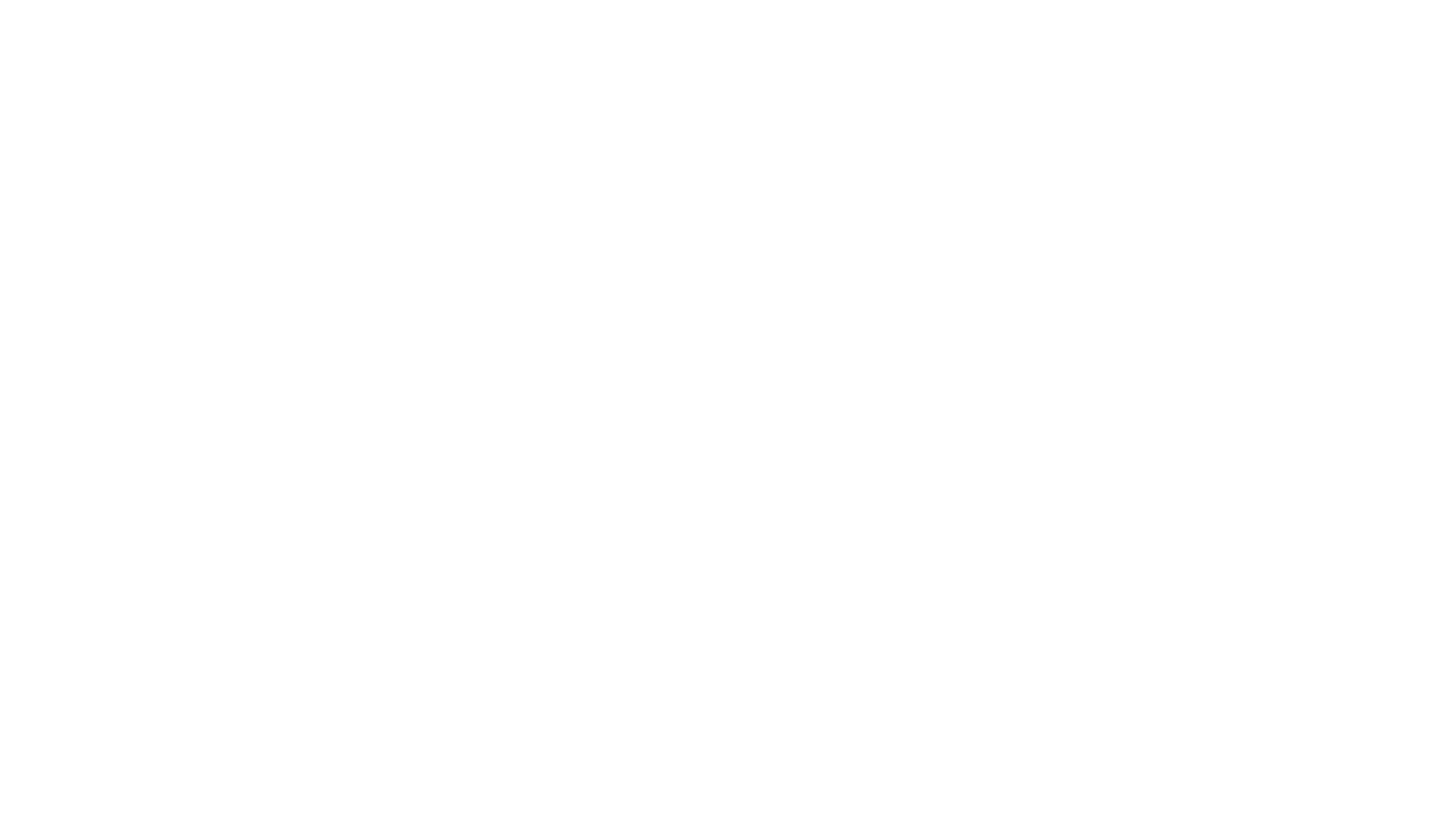

Supplement: S1 Data — (ZIP) [file ppat.1014384.s005.zip › gsdmd_western blot/gsdmd_western blot/GD_cleavage E1/gd_cleavage.pptx]
