# Supplementary figures and images for: Inflammasome activation dictates the efficacy of antimycobacterial activity of frontline TB drugs
Source: PLoS Pathog. 2026 Jul 16;22(7):e1014384. doi: 10.1371/journal.ppat.1014384 (PMC13399518; doi:10.1371/journal.ppat.1014384)

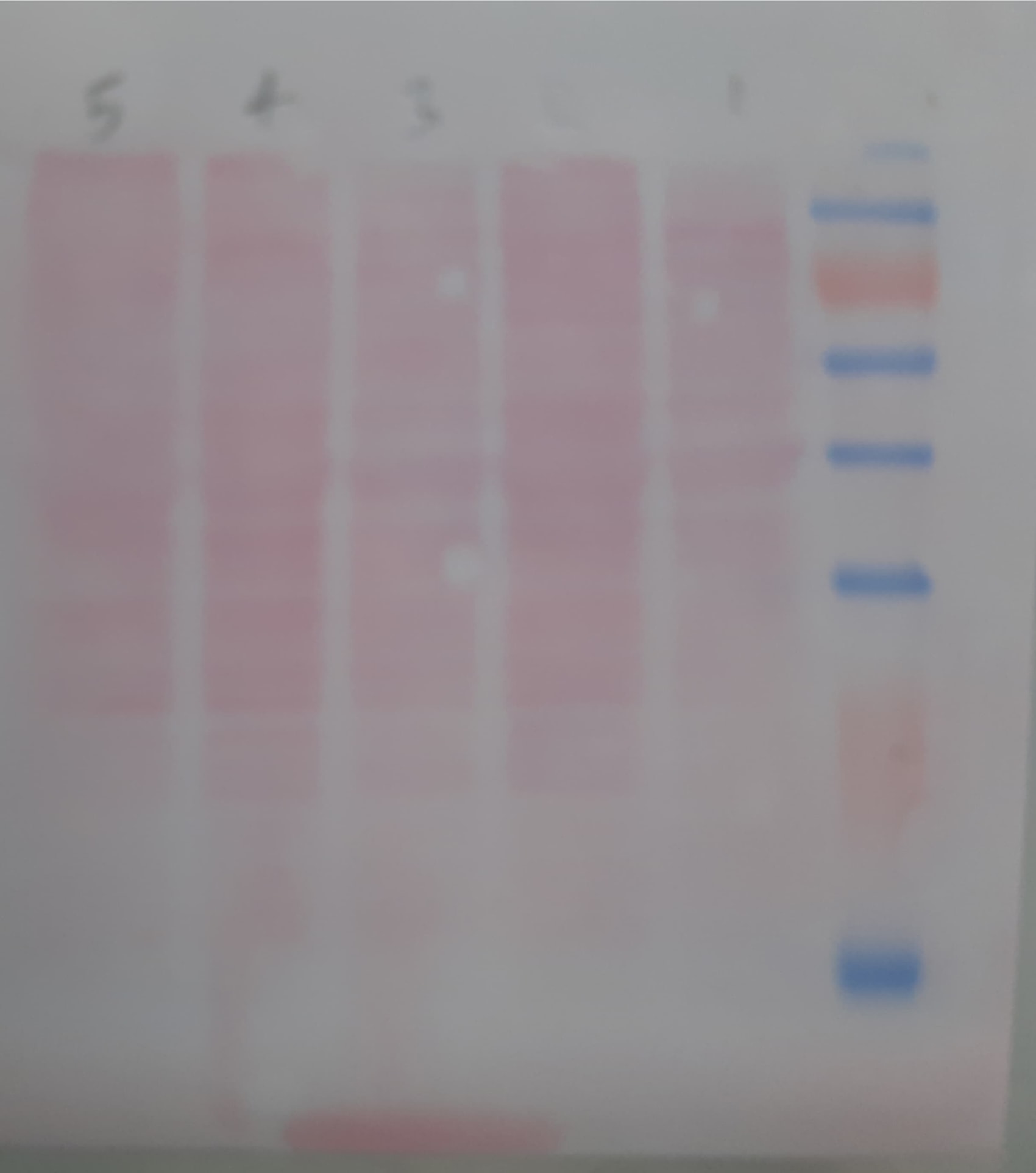

Supplement: S1 Data — (ZIP) [file ppat.1014384.s005.zip › gsdmd_western blot/gsdmd_western blot/GD_cleavage E1/gsdmd 25 Jul 2023_1.jpg]

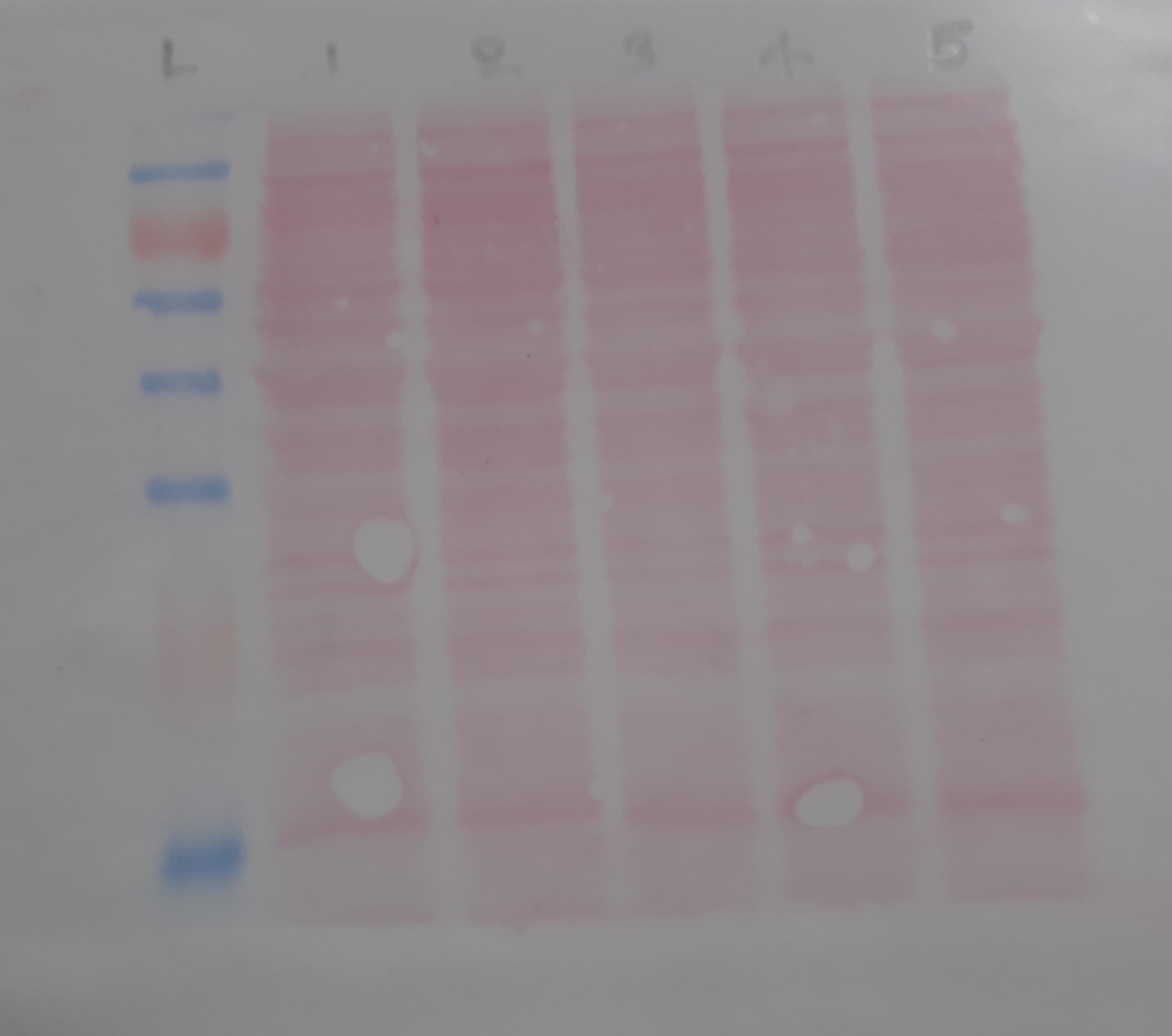

Supplement: S1 Data — (ZIP) [file ppat.1014384.s005.zip › gsdmd_western blot/gsdmd_western blot/GD_cleavage E3/Adobe Scan 10 Jul 2023 (1)_page-0001.jpg]

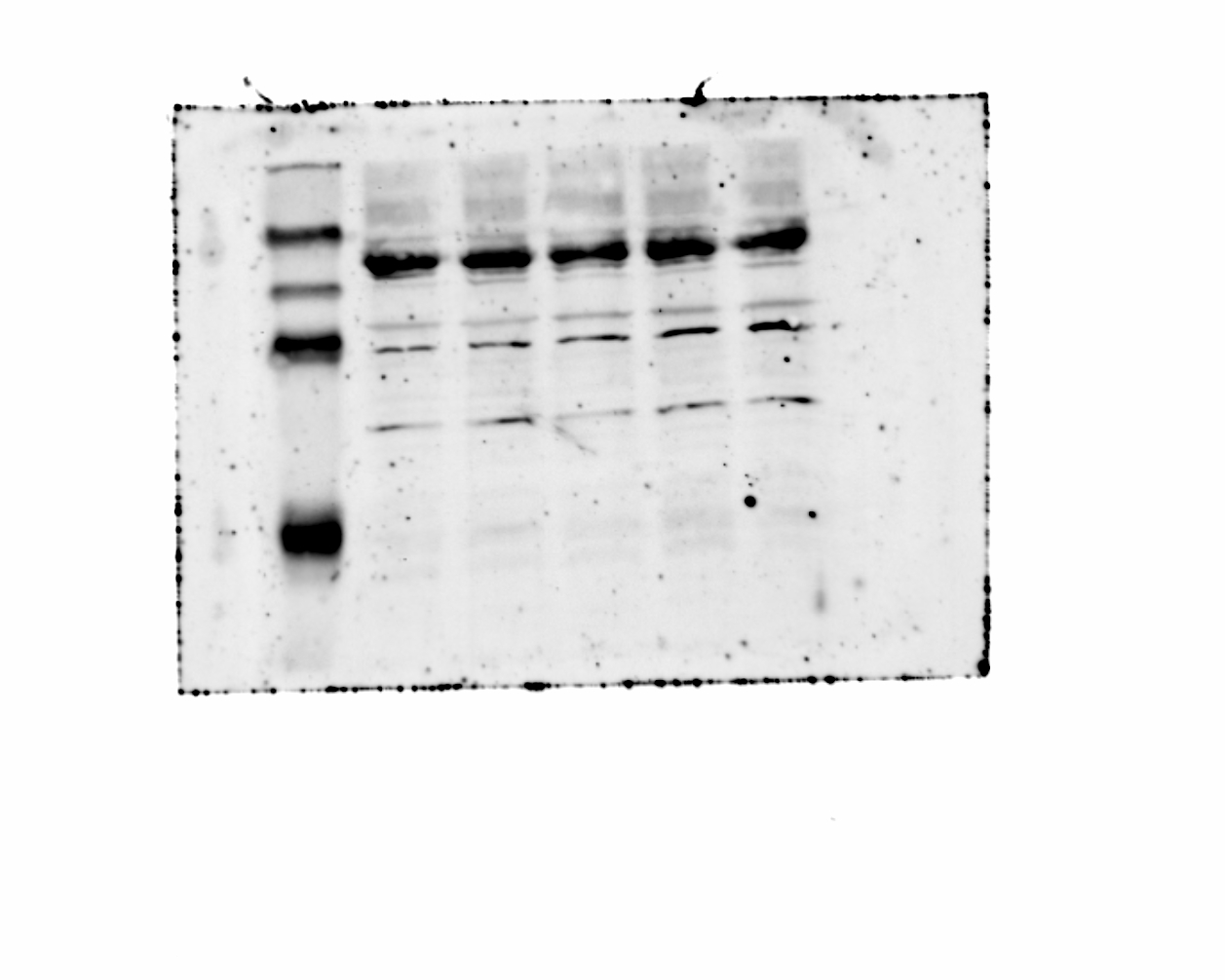

Supplement: S1 Data — (ZIP) [file ppat.1014384.s005.zip › gsdmd_western blot/gsdmd_western blot/GD_cleavage_E2/DEMO 2024-02-26 10h24m13s durgesh(IRDye 800CW).jpg]

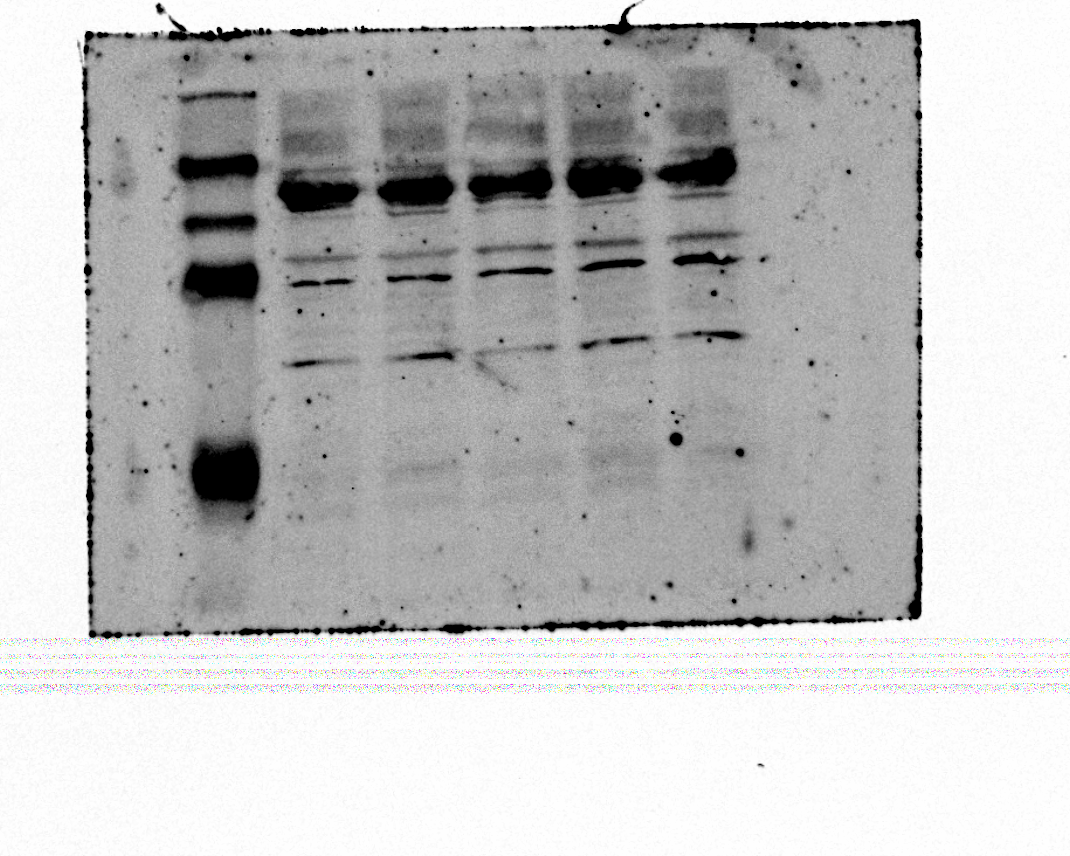

Supplement: S1 Data — (ZIP) [file ppat.1014384.s005.zip › gsdmd_western blot/gsdmd_western blot/GD_cleavage_E2/DEMO 2024-02-26 10h27m01s durgesh(IRDye 800CW).tif]

## Slide 1
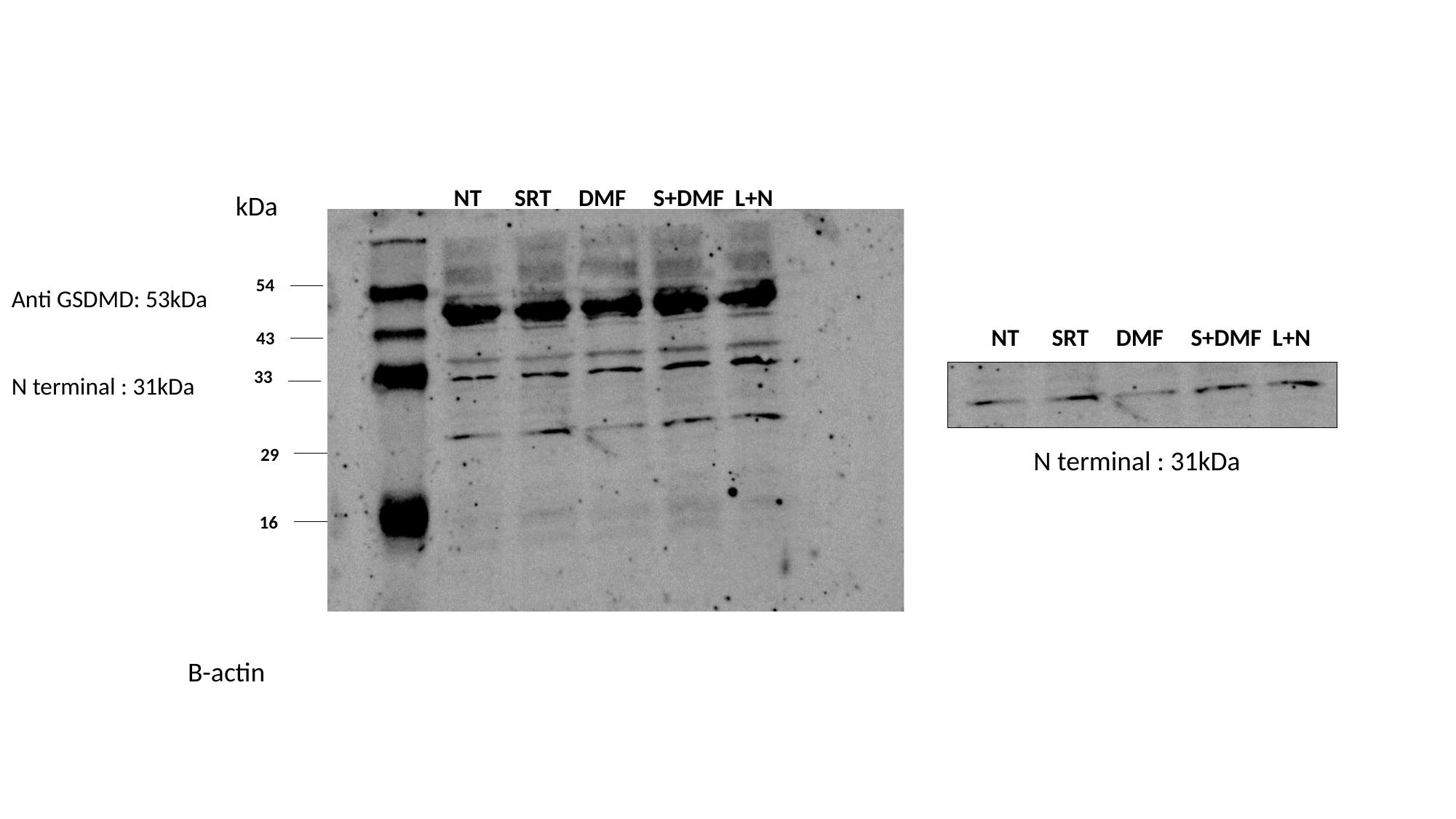

NT SRT DMF S+DMF L+N
kDa
54
Anti GSDMD: 53kDa
N terminal : 31kDa
 NT SRT DMF S+DMF L+N
43
33
29
N terminal : 31kDa
16
B-actin

Supplement: S1 Data — (ZIP) [file ppat.1014384.s005.zip › gsdmd_western blot/gsdmd_western blot/GD_cleavage_E2/gd.pptx]

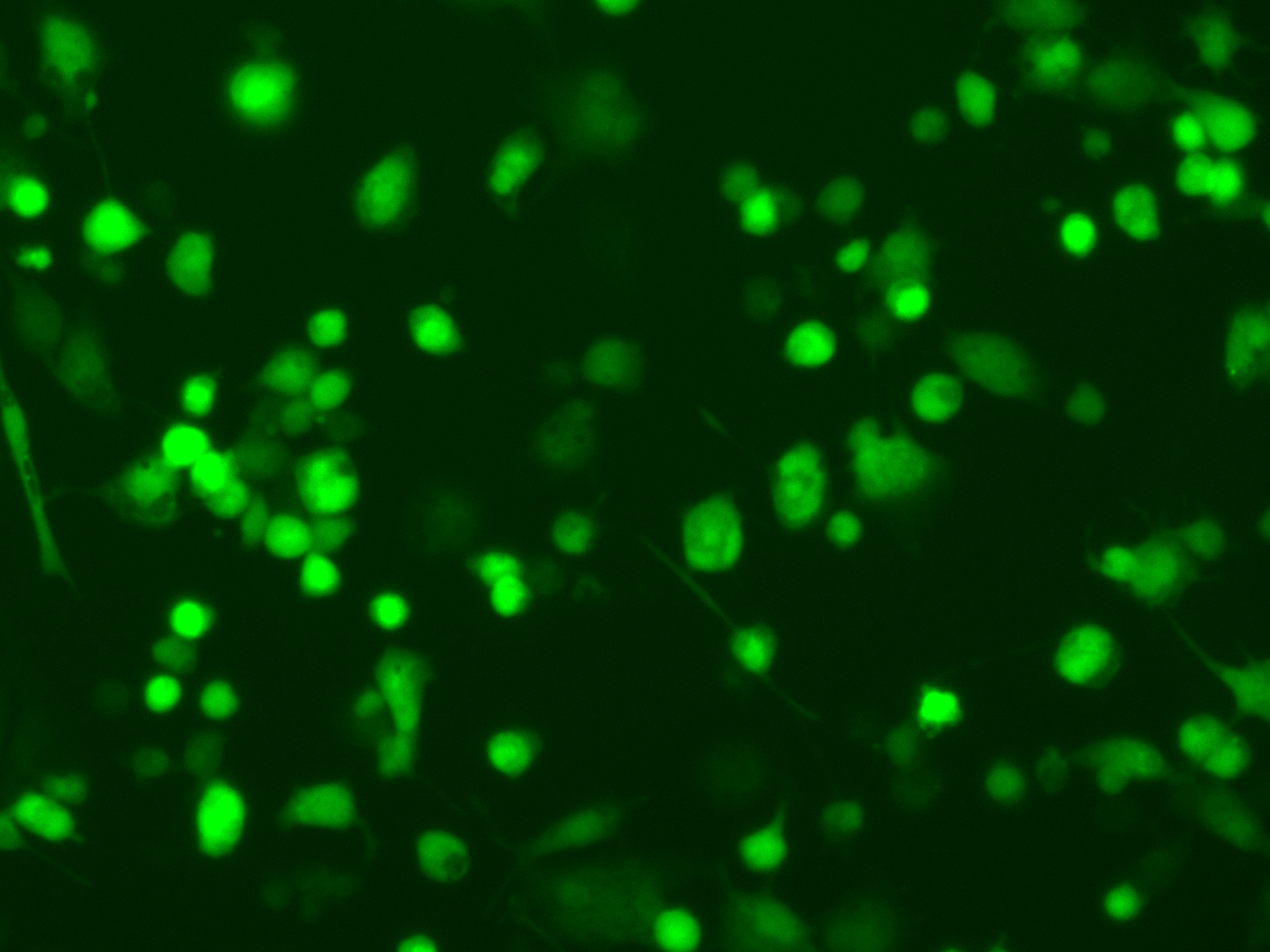

Supplement: S2 Data — (ZIP) [file ppat.1014384.s006.zip › IPG4AM/nt_0005_GFP.jpg]

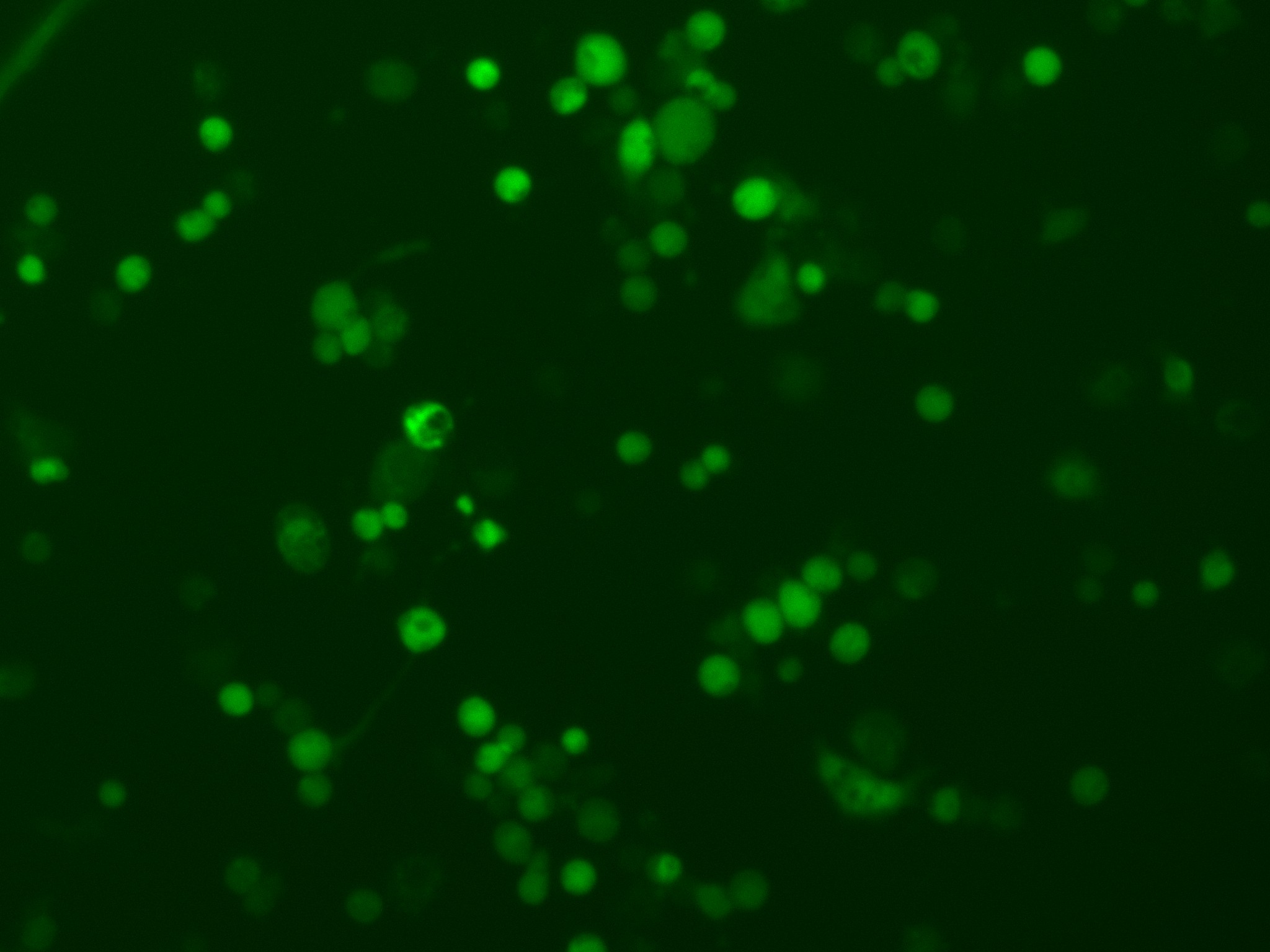

Supplement: S2 Data — (ZIP) [file ppat.1014384.s006.zip › IPG4AM/s+dmf_0003_GFP.jpg]

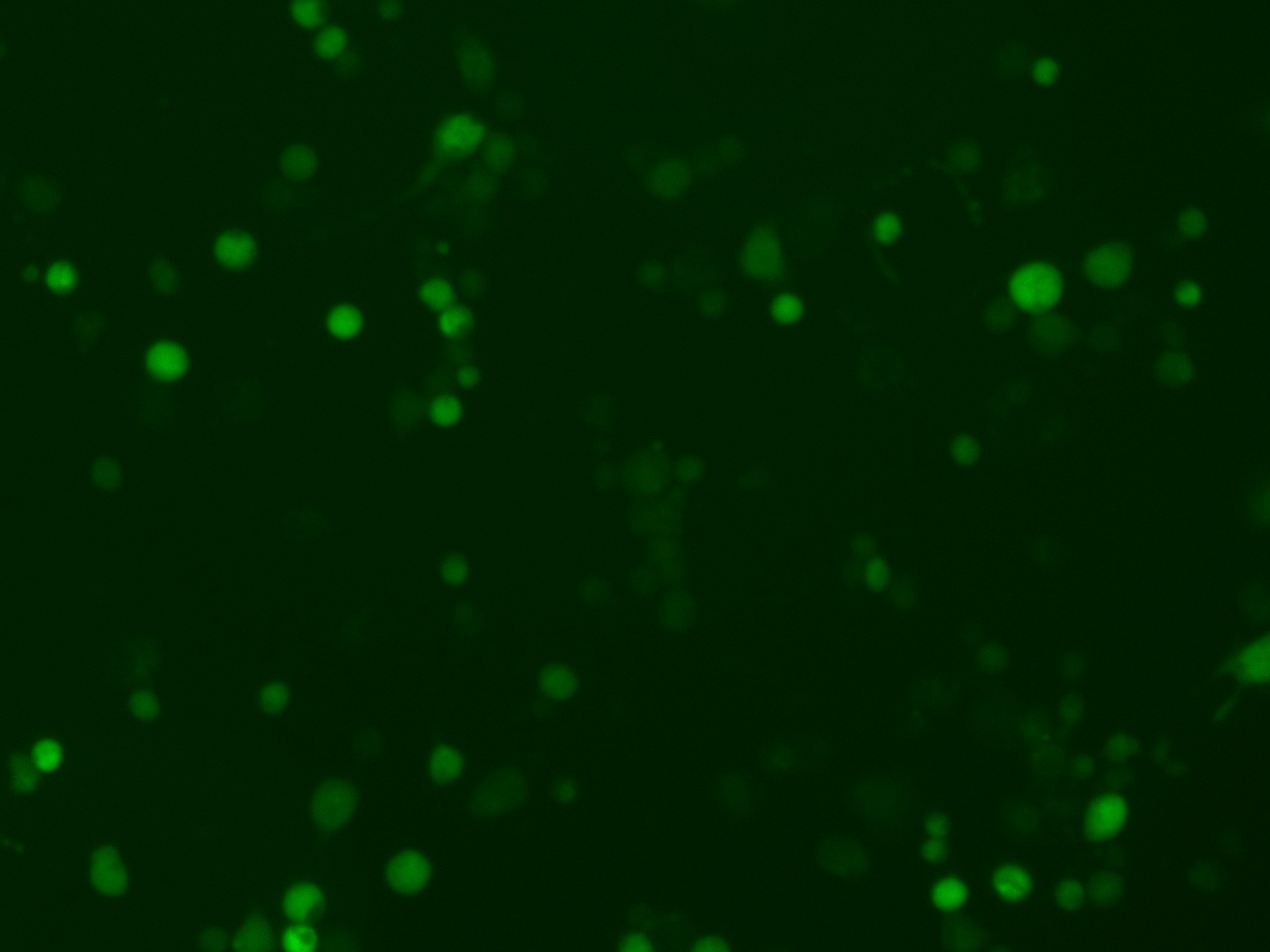

Supplement: S2 Data — (ZIP) [file ppat.1014384.s006.zip › IPG4AM/srt_0001_GFP.jpg]

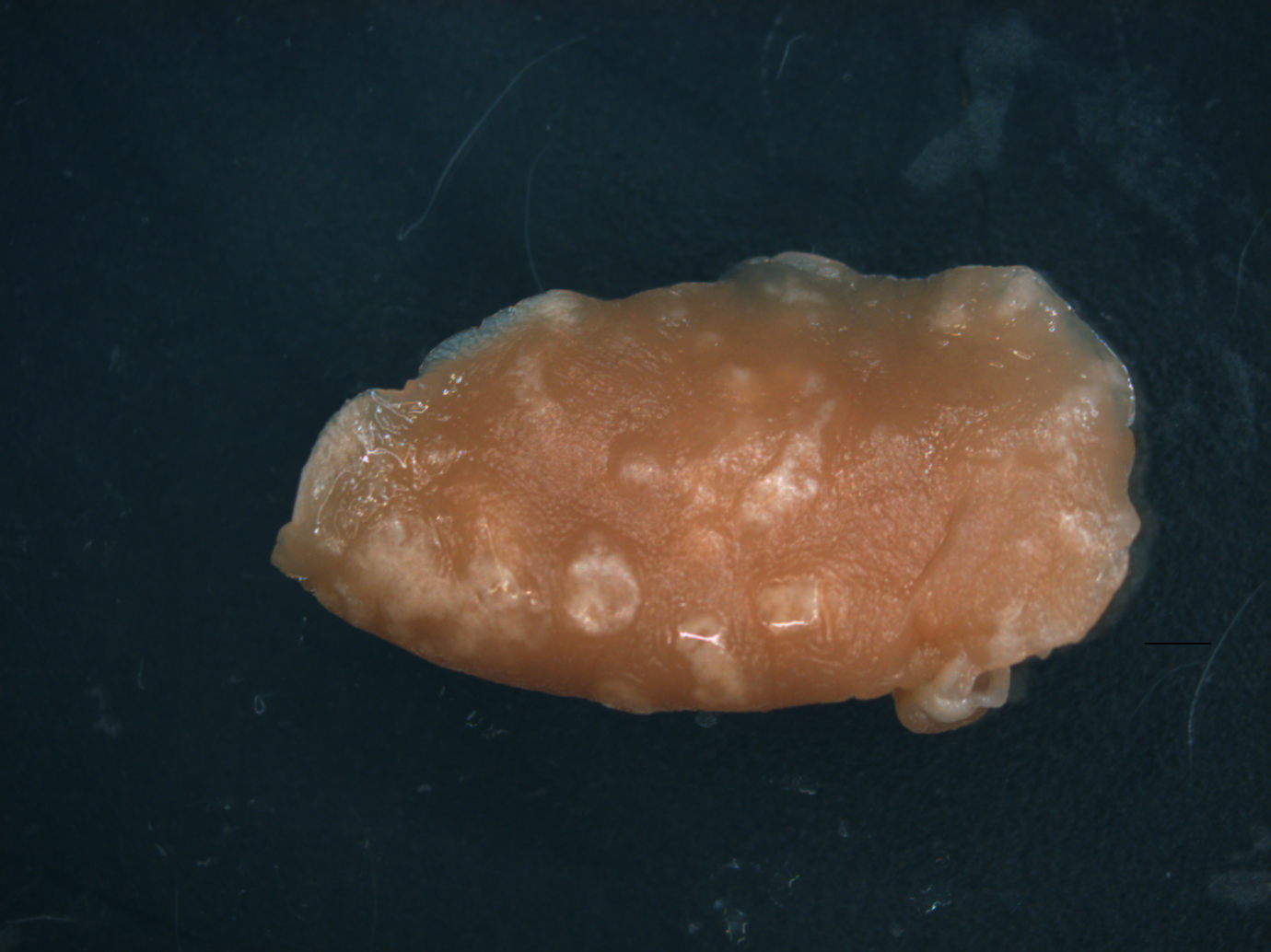

Supplement: S3 Data — (ZIP) [file ppat.1014384.s007.zip › lung gross/T1hrzes-1.tif]

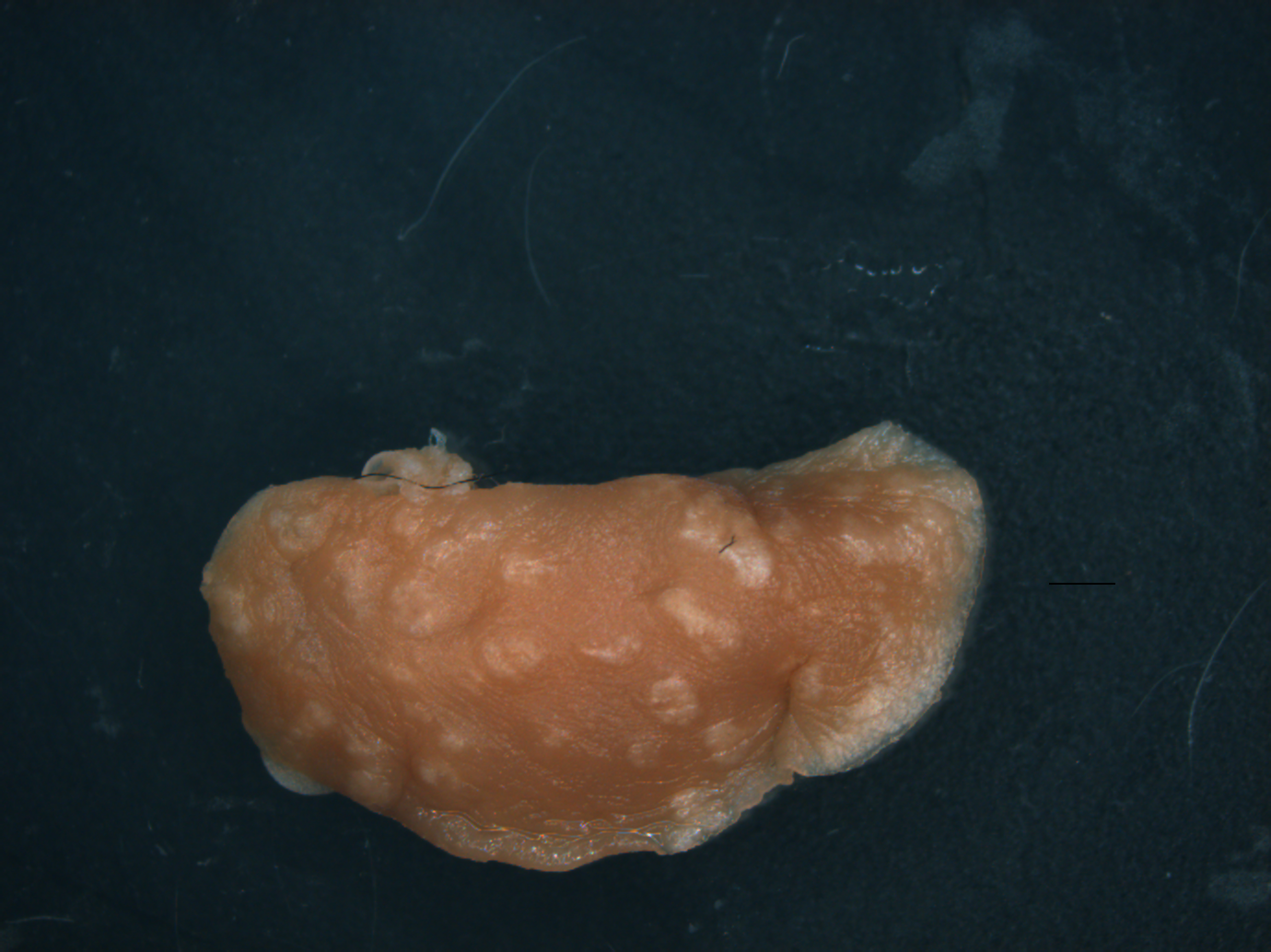

Supplement: S3 Data — (ZIP) [file ppat.1014384.s007.zip › lung gross/T1hrzes-2.tif]

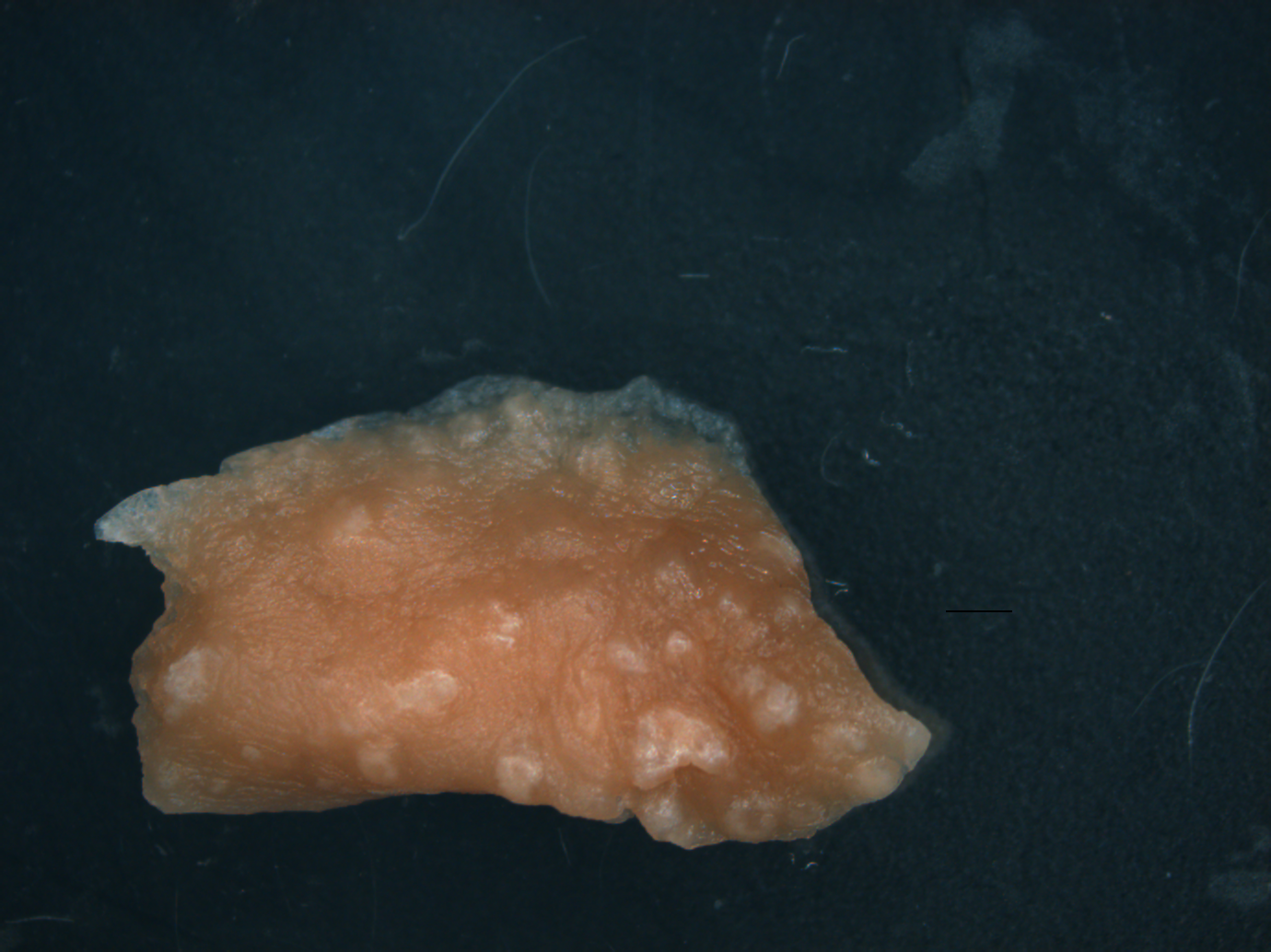

Supplement: S3 Data — (ZIP) [file ppat.1014384.s007.zip › lung gross/T1hrzes-3.tif]

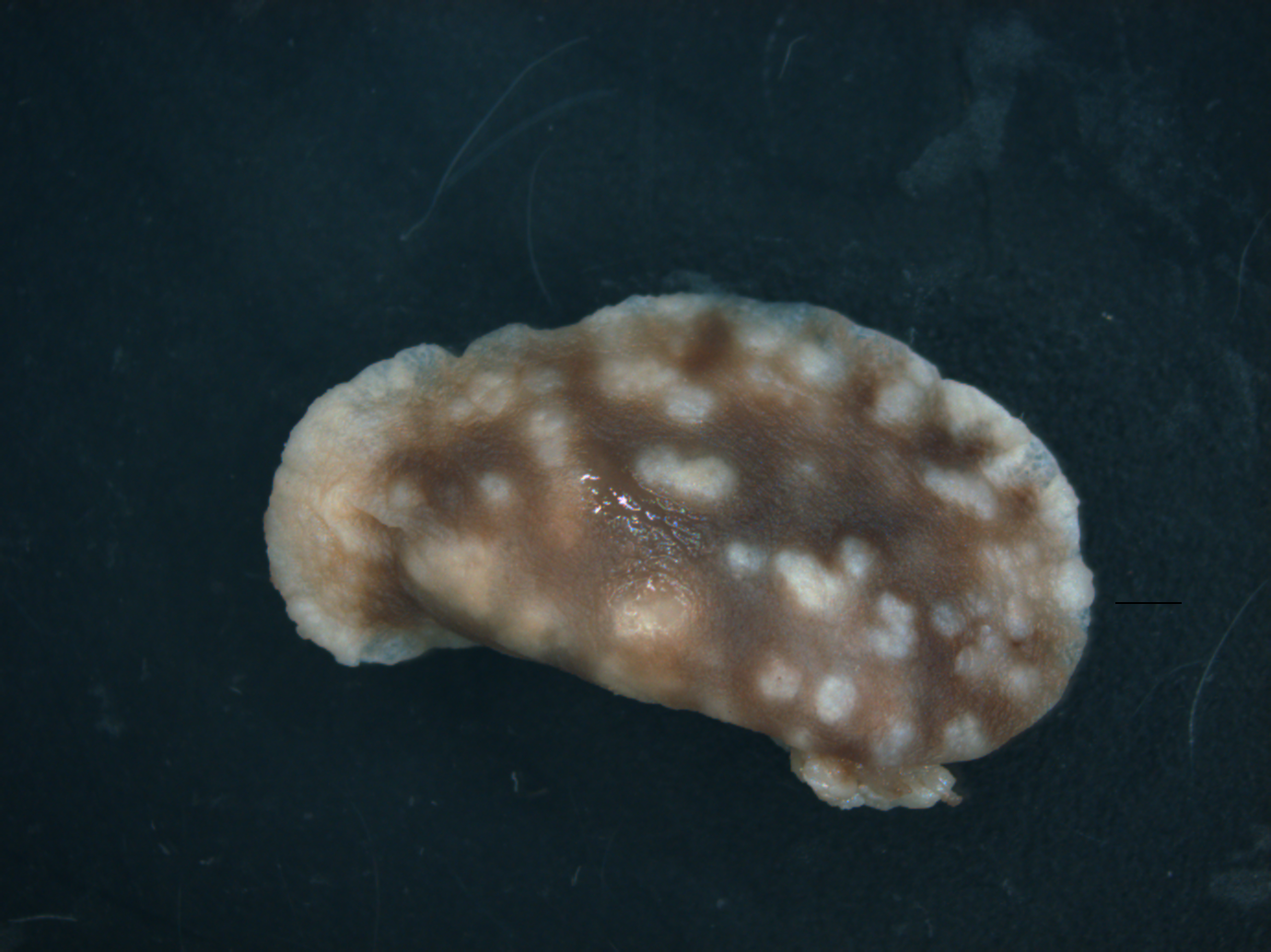

Supplement: S3 Data — (ZIP) [file ppat.1014384.s007.zip › lung gross/T1hrzesd-2.tif]

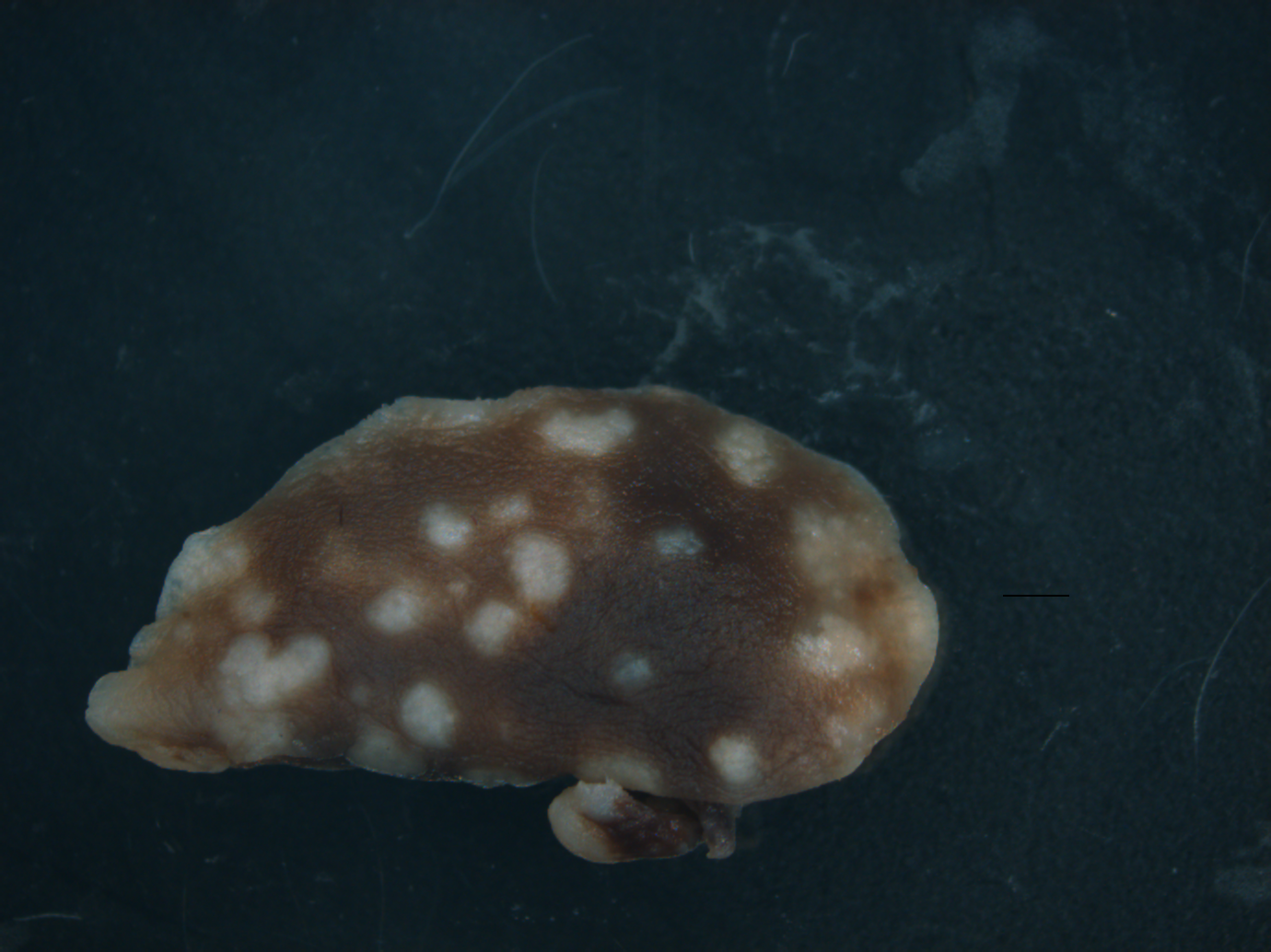

Supplement: S3 Data — (ZIP) [file ppat.1014384.s007.zip › lung gross/T1hrzesd-3.tif]

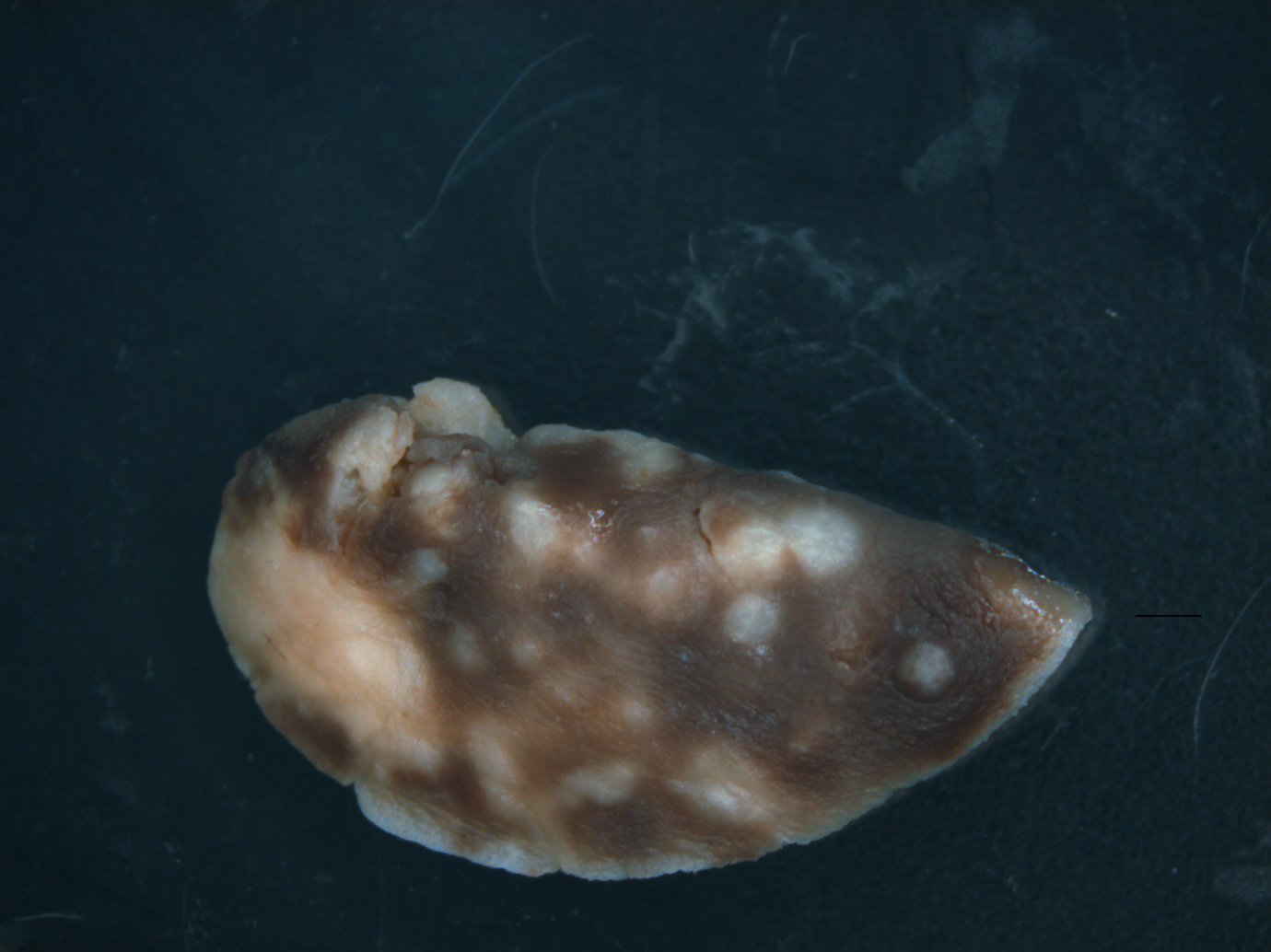

Supplement: S3 Data — (ZIP) [file ppat.1014384.s007.zip › lung gross/T1hrzesd-4.tif]

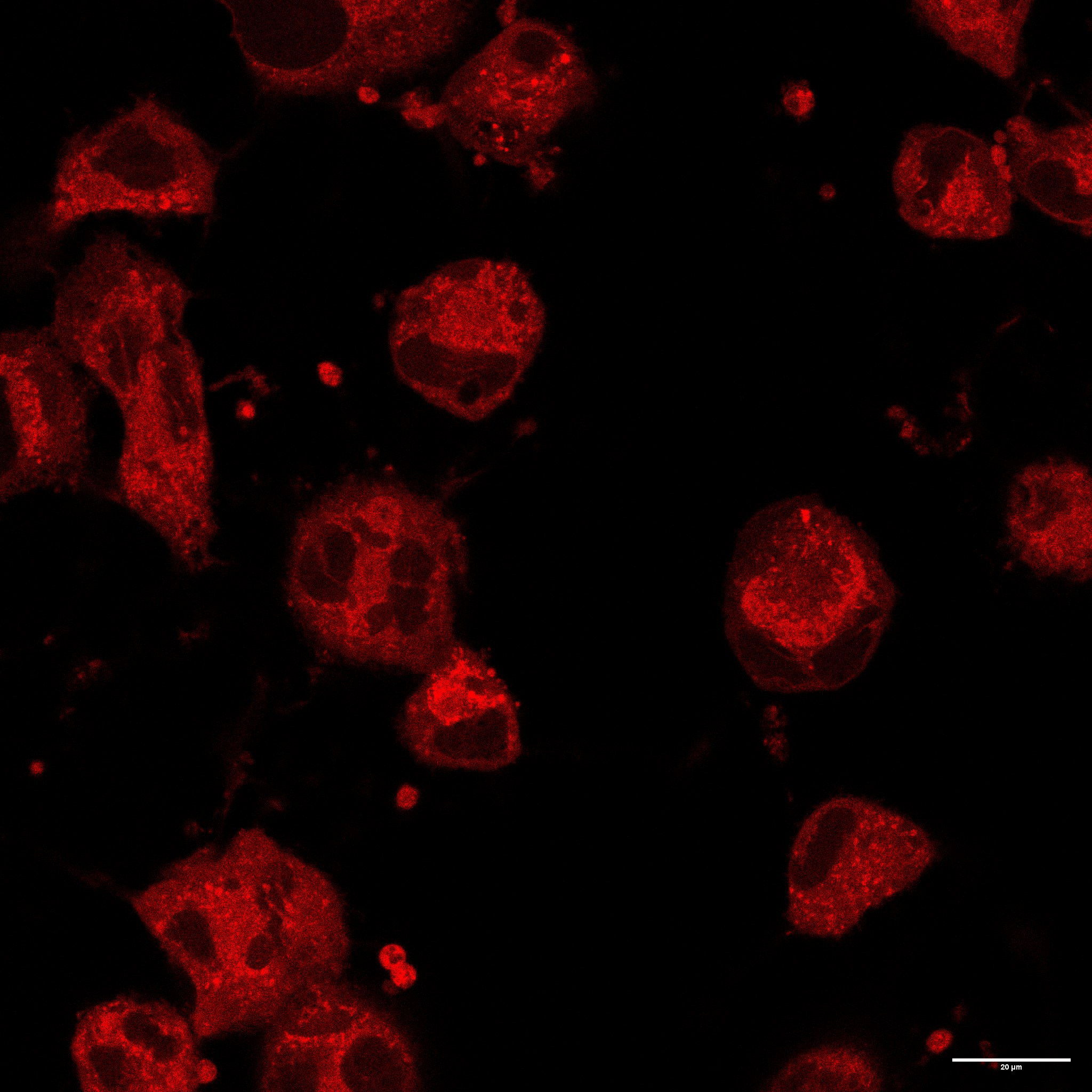

Supplement: S4 Data — (ZIP) [file ppat.1014384.s008.zip › mito red/nt 6H.lif - Image005.jpg]

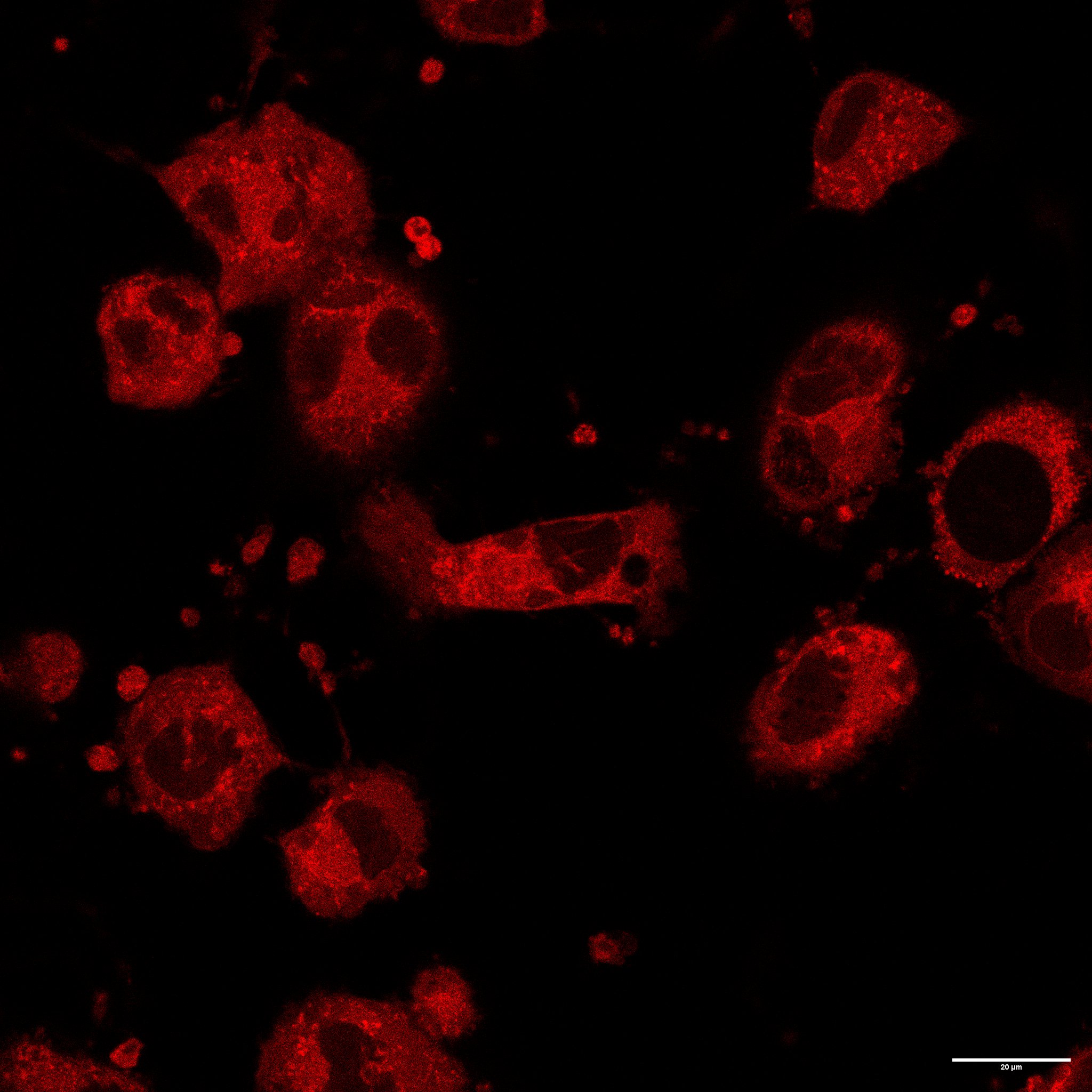

Supplement: S4 Data — (ZIP) [file ppat.1014384.s008.zip › mito red/nt 6H.lif - Image008.jpg]

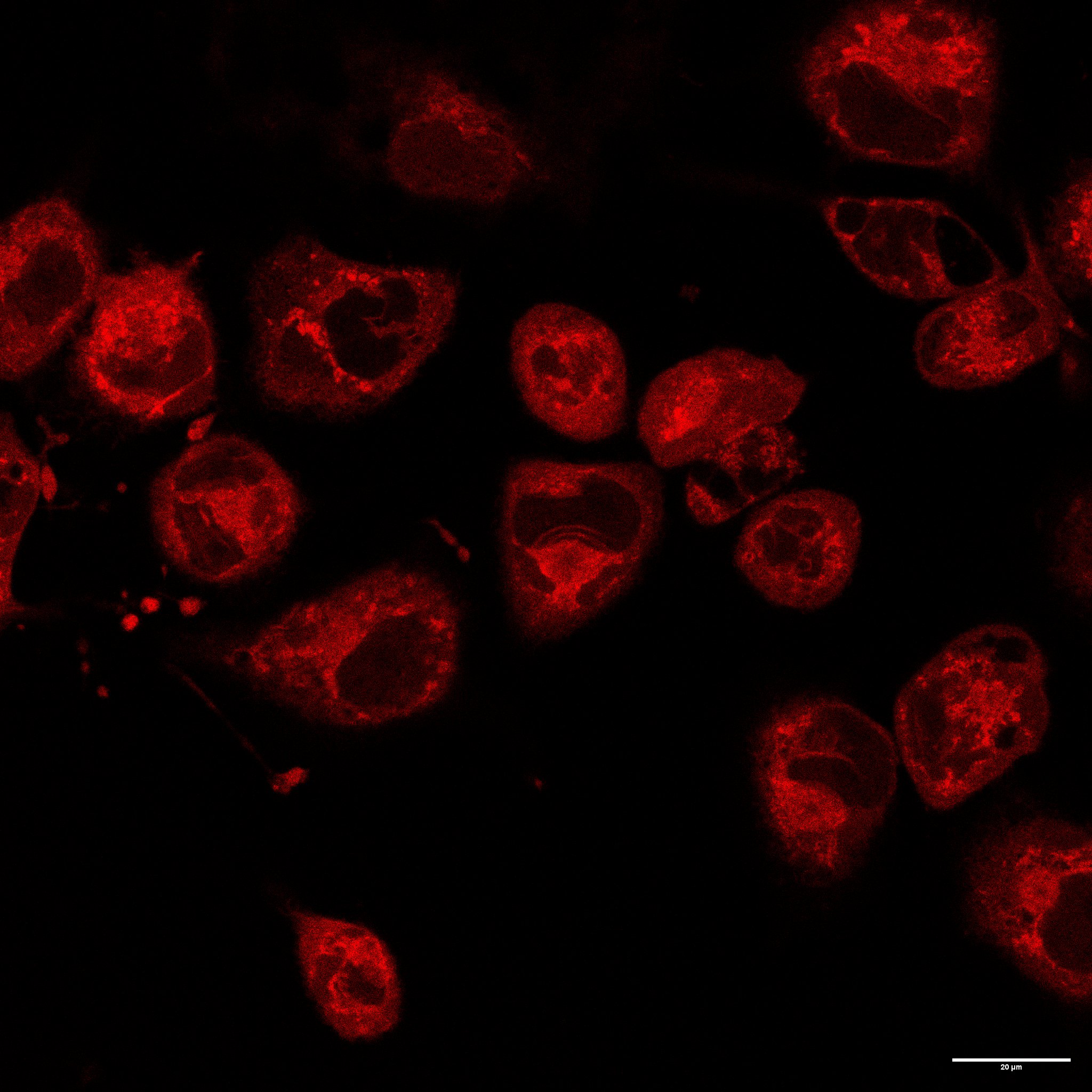

Supplement: S4 Data — (ZIP) [file ppat.1014384.s008.zip › mito red/NT24H.lif - Image008.jpg]

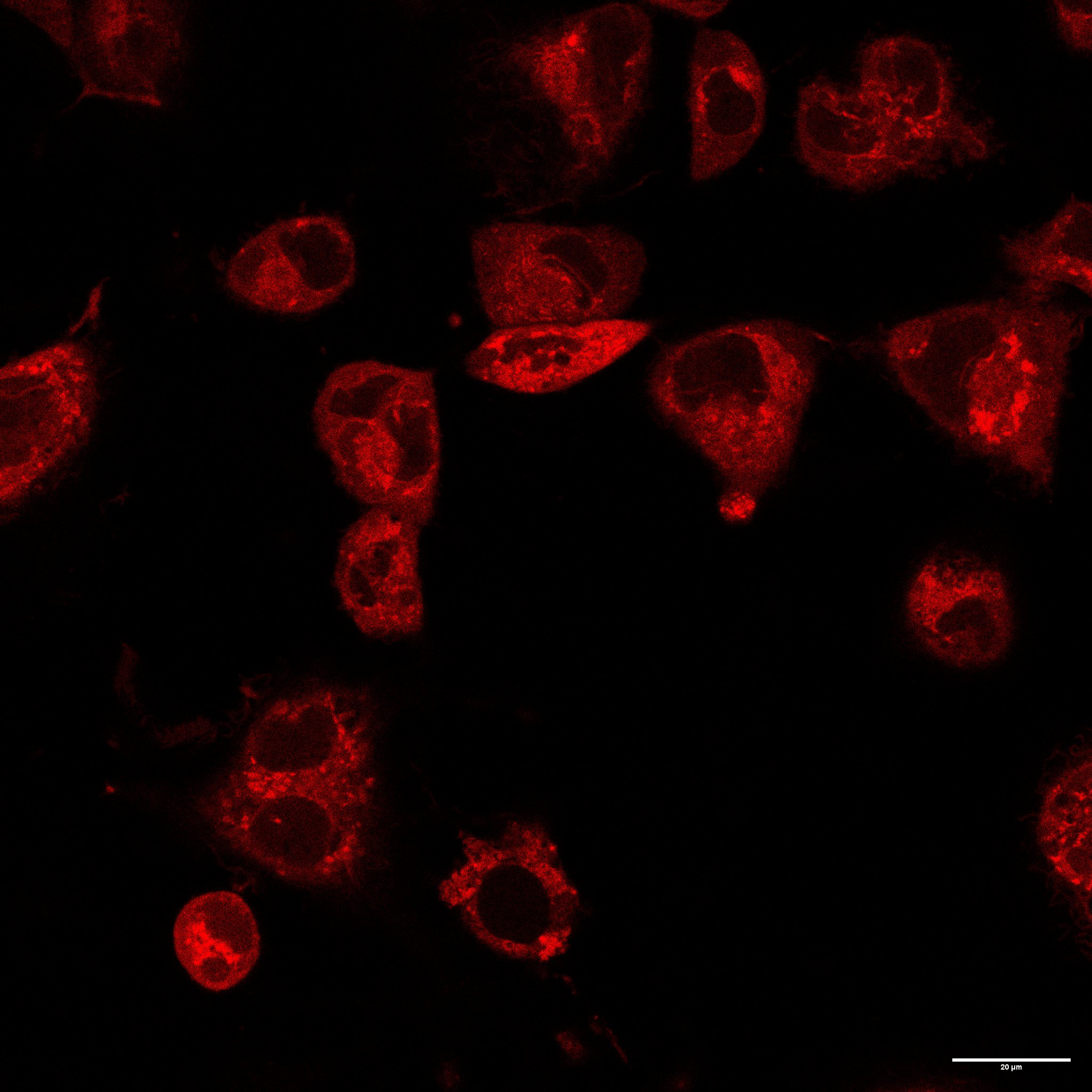

Supplement: S4 Data — (ZIP) [file ppat.1014384.s008.zip › mito red/NT24H.lif - Image009.jpg]

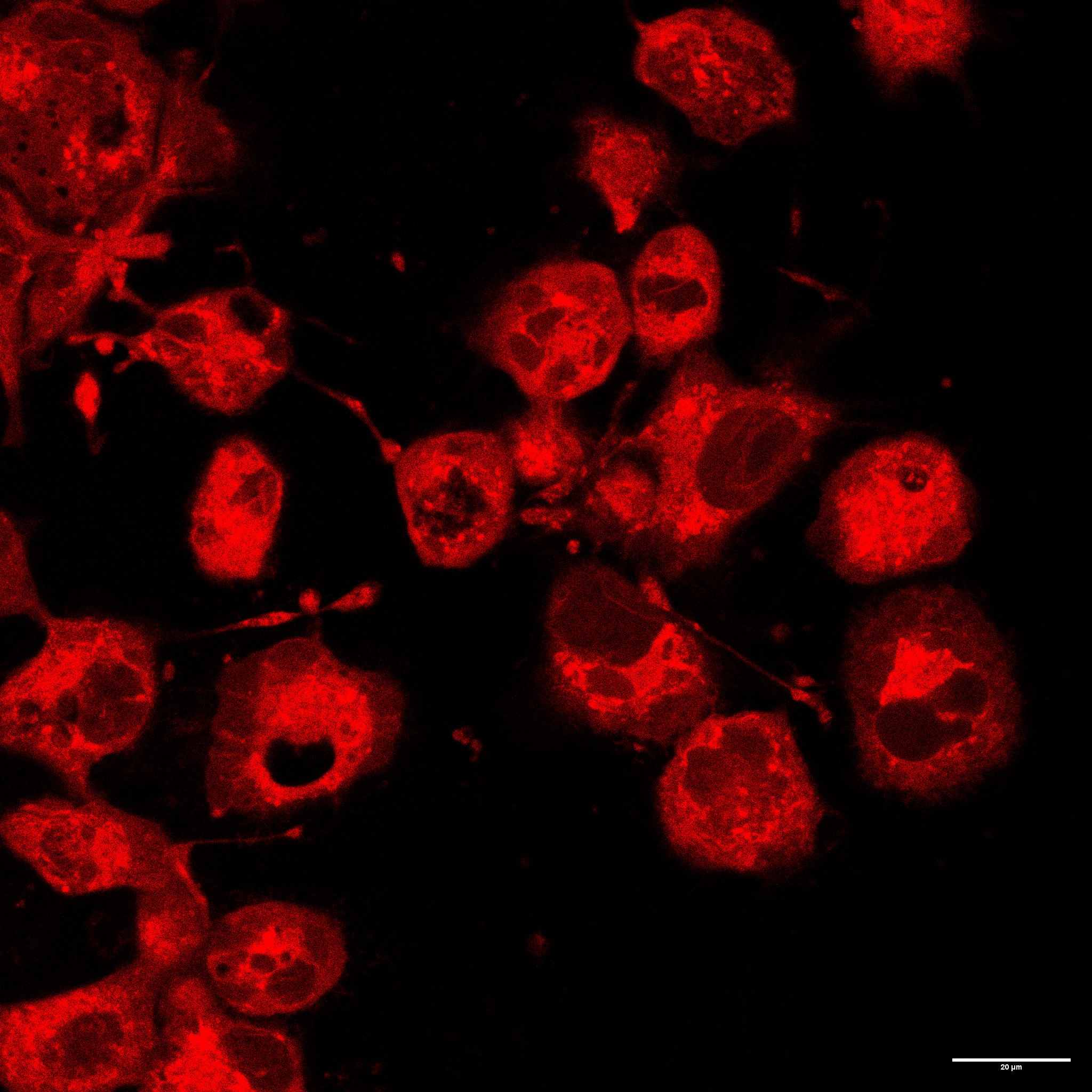

Supplement: S4 Data — (ZIP) [file ppat.1014384.s008.zip › mito red/srt 6H.lif - Image005.jpg]

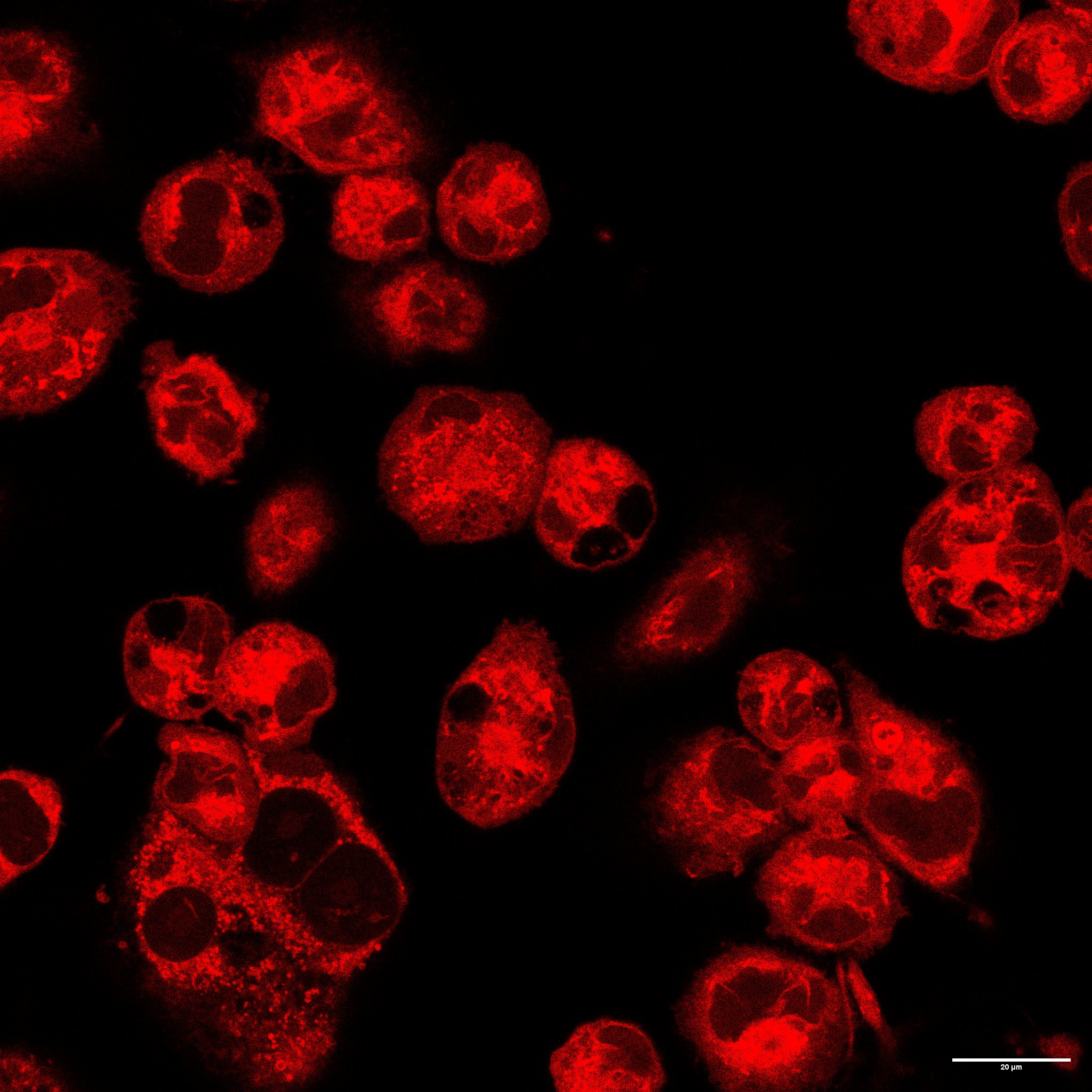

Supplement: S4 Data — (ZIP) [file ppat.1014384.s008.zip › mito red/srt 6H.lif - Image010.jpg]

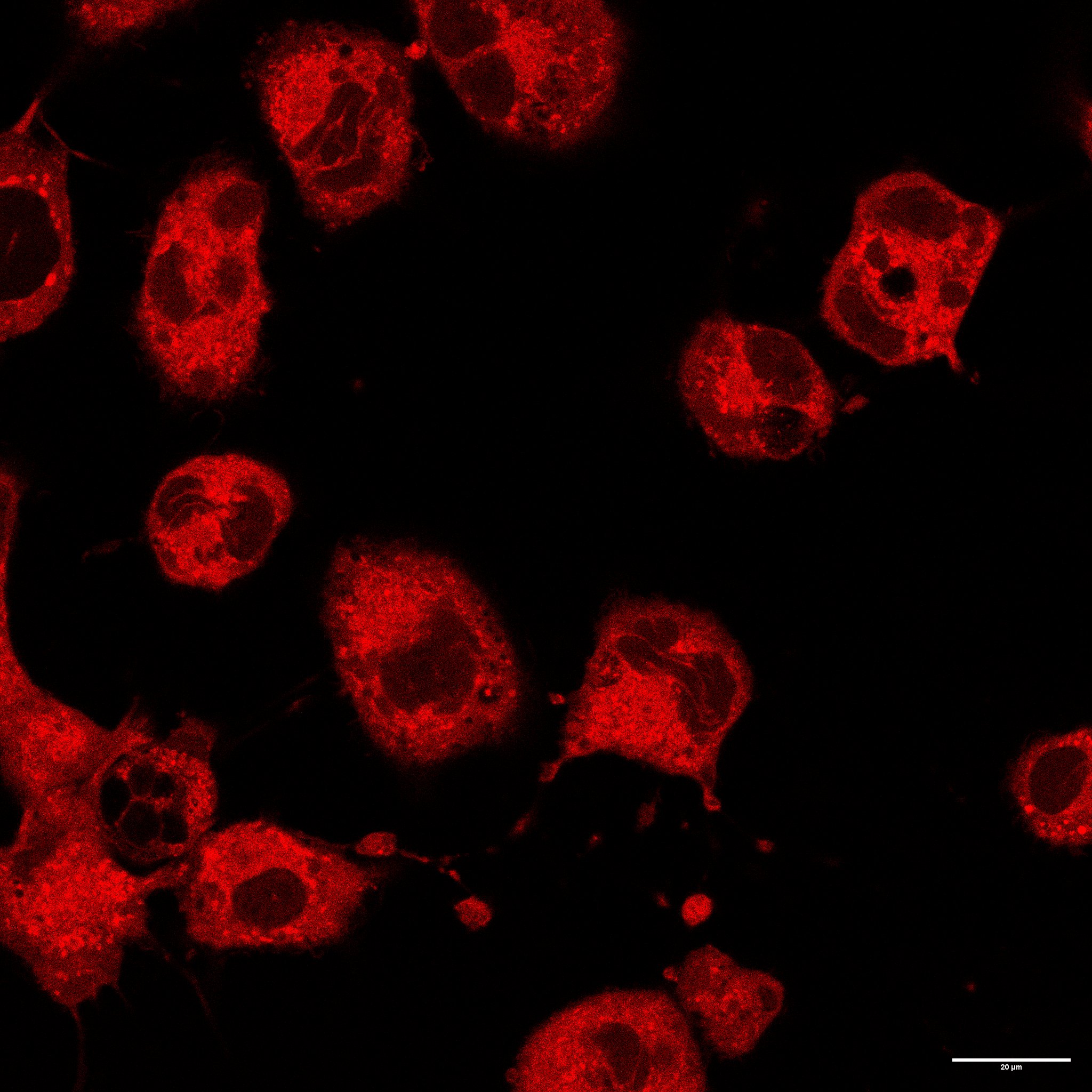

Supplement: S4 Data — (ZIP) [file ppat.1014384.s008.zip › mito red/SRT24H.lif - Image009.jpg]

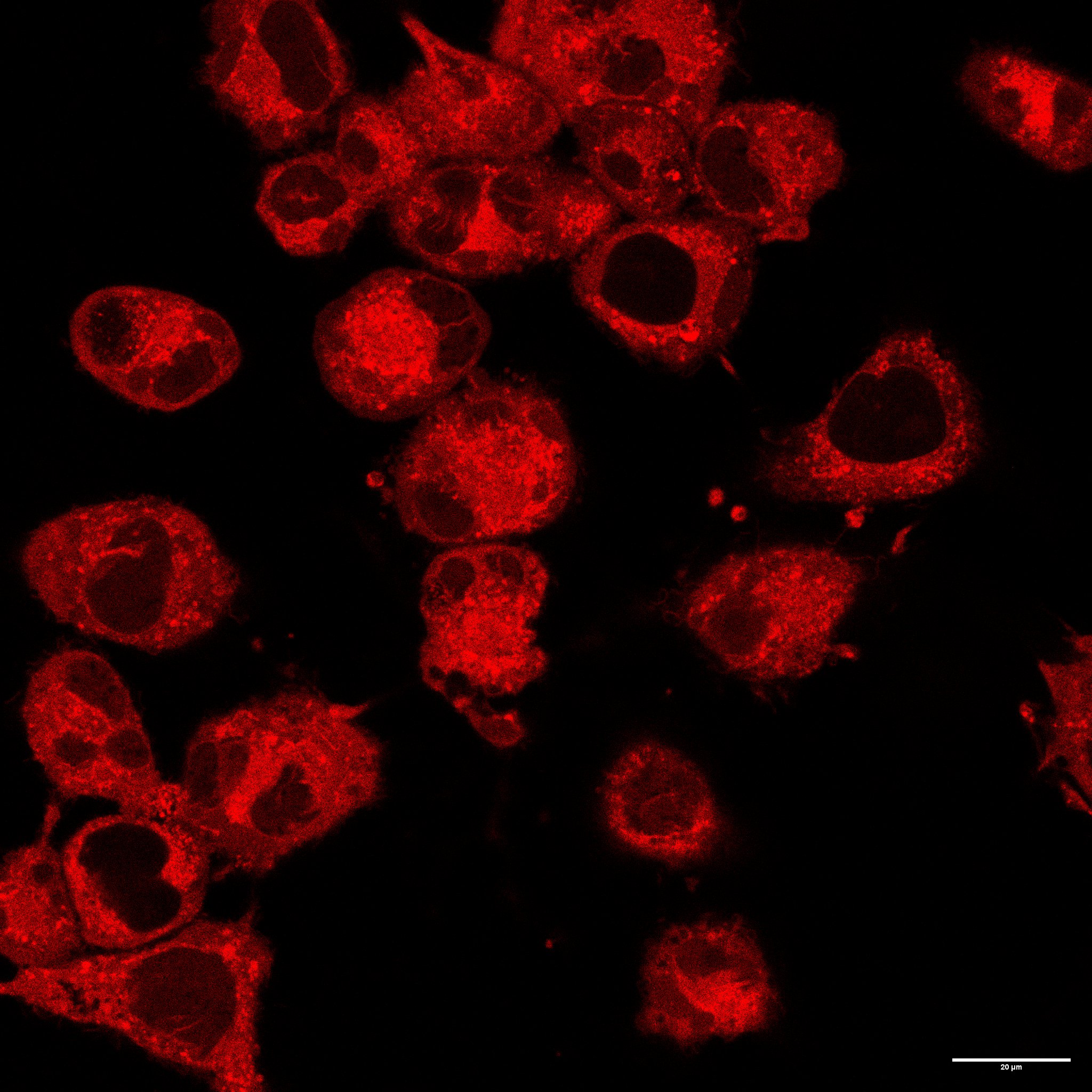

Supplement: S4 Data — (ZIP) [file ppat.1014384.s008.zip › mito red/SRT24H.lif - Image010.jpg]
